# Phenotypic Plasticity through Transcriptional Regulation of the Evolutionary Hotspot Gene *tan* in *Drosophila melanogaster*

**DOI:** 10.1371/journal.pgen.1006218

**Published:** 2016-08-10

**Authors:** Jean-Michel Gibert, Emmanuèle Mouchel-Vielh, Sandra De Castro, Frédérique Peronnet

**Affiliations:** Sorbonne Universités, Université Pierre et Marie Curie (UPMC), CNRS, Institut de Biologie Paris-Seine (IBPS), Laboratoire de Biologie du Développement, Equipe “Contrôle épigénétique de l’homéostasie et de la plasticité du développement”, Paris, France; University of California Davis, UNITED STATES

## Abstract

Phenotypic plasticity is the ability of a given genotype to produce different phenotypes in response to distinct environmental conditions. Phenotypic plasticity can be adaptive. Furthermore, it is thought to facilitate evolution. Although phenotypic plasticity is a widespread phenomenon, its molecular mechanisms are only beginning to be unravelled. Environmental conditions can affect gene expression through modification of chromatin structure, mainly *via* histone modifications, nucleosome remodelling or DNA methylation, suggesting that phenotypic plasticity might partly be due to chromatin plasticity. As a model of phenotypic plasticity, we study abdominal pigmentation of *Drosophila melanogaster* females, which is temperature sensitive. Abdominal pigmentation is indeed darker in females grown at 18°C than at 29°C. This phenomenon is thought to be adaptive as the dark pigmentation produced at lower temperature increases body temperature. We show here that temperature modulates the expression of *tan* (*t*), a pigmentation gene involved in melanin production. *t* is expressed 7 times more at 18°C than at 29°C in female abdominal epidermis. Genetic experiments show that modulation of *t* expression by temperature is essential for female abdominal pigmentation plasticity. Temperature modulates the activity of an enhancer of *t* without modifying compaction of its chromatin or level of the active histone mark H3K27ac. By contrast, the active mark H3K4me3 on the *t* promoter is strongly modulated by temperature. The H3K4 methyl-transferase involved in this process is likely Trithorax, as we show that it regulates *t* expression and the H3K4me3 level on the *t* promoter and also participates in female pigmentation and its plasticity. Interestingly, *t* was previously shown to be involved in inter-individual variation of female abdominal pigmentation in *Drosophila melanogaster*, and in abdominal pigmentation divergence between *Drosophila* species. Sensitivity of *t* expression to environmental conditions might therefore give more substrate for selection, explaining why this gene has frequently been involved in evolution of pigmentation.

## Author Summary

Environmental conditions can strongly modulate the phenotype produced by a particular genotype. This process, called phenotypic plasticity, has major implications in medicine and agricultural sciences, and is thought to facilitate evolution. Phenotypic plasticity is observed in many animals and plants but its mechanisms are only partially understood. As a model of phenotypic plasticity, we study the effect of temperature on female abdominal pigmentation in the fruit fly *Drosophila melanogaster*. Here we show that temperature affects female abdominal pigmentation by modulating the expression of *tan* (*t*), a gene involved in melanin production, in female abdominal epidermis. This effect is mediated at least partly by a particular regulatory sequence of *t*, the *t_MSE* enhancer. However we detected no modulation of chromatin structure of *t_MSE* by temperature. By contrast, the level of the active chromatin mark H3K4me3 on the *t* promoter is strongly increased at lower temperature. We show that the H3K4 methyl-transferase Trithorax is involved in female abdominal pigmentation and its plasticity and regulates *t* expression and H3K4me3 level on the *t* promoter. Several studies have linked *t* to pigmentation evolution within and between *Drosophila* species. Our results suggest that sensitivity of *t* expression to temperature might facilitate its role in pigmentation evolution.

## Introduction

Phenotypic plasticity, “the property of a given genotype to produce different phenotypes in response to distinct environmental conditions” [1], is a widespread phenomenon. Phenotypic plasticity can be adaptive if different but optimal phenotypes are produced by a given genotype in distinct environments [2]. Furthermore, phenotypic plasticity could facilitate evolution [3–6]. In particular, Conrad Waddington showed that changes in environmental conditions can reveal cryptic genetic variation that can be selected, allowing to fix a phenotype initially observed only in particular environmental conditions [7,8]. Waddington called this process “genetic assimilation”. Analysis of phenotypic plasticity and morphological complexity in an evolutionary framework supports indeed the idea that phenotypic plasticity increases evolutionary potential. For example, a recent study on feeding structure evolution in nematods revealed that phenotypic plasticity correlates with morphological diversification [9]. The question then arises whether the same genes are involved in phenotypic plasticity and in phenotypic variation within and between species. To address this question, the molecular mechanisms underlying phenotypic plasticity need to be identified. Several examples show that environmental factors can strongly affect the transcriptome [10] through modification of chromatin structure by DNA methylation [11], histone mark apposition [12] or nucleosome remodelling [13]. In *Drosophila melanogaster*, female abdominal pigmentation is a plastic trait as it is darker in females grown at 18°C than at 29°C [14]. As low temperature leads to darker pigmentation, which increases body temperature, the thermal plasticity of female abdominal pigmentation is thought to be adaptive [14]. Abdominal pigmentation in drosophilids is a particularly appropriate model to study phenotypic plasticity, as the genes involved in abdominal pigmentation are well known. Indeed, abdominal pigmentation has been used as a model to dissect the genetic bases of sexual dimorphism and of variation within or between species [15–23]. In none of these studies, which focussed on genetic factors and were performed in standard conditions (usually at 25°C), was the effect of the environment taken into account. However, *Drosophila melanogaster* can develop between 12°C and 30°C [24]. As temperature varies spatially and temporally in the wild, taking it into account is paramount to understand the development and evolution of abdominal pigmentation. Using mainly genetics approaches, we previously showed that temperature acts on melanin production by modulating a chromatin regulator network, but we did not further dissect the underlying molecular mechanisms [25]. Here, we identify the pigmentation gene *tan (t)* as the major structural gene involved in female abdominal pigmentation plasticity and we show that chromatin structure at this *locus* is modulated by temperature. Temperature dramatically modulates *t* expression in the female abdominal epidermis and this modulation plays a major role in female abdominal pigmentation plasticity. Temperature modulates the activity of an enhancer of *t*, *t_MSE* [17], but had no detectable effect on its chromatin structure. By contrast, the active histone mark H3K4me3 is strongly enriched on the *t* promoter at low temperature. The H3K4 methyl-transferase responsible for this effect is likely Trithorax (Trx). Indeed, we show that Trx regulates *t* expression and the level of H3K4me3 on the *t* promoter, and is involved in abdominal pigmentation as well as in its plasticity. As *t* has been linked to pigmentation divergence within or between *Drosophila species* [17,19,20,26], *t* is listed among hotspot *loci* of evolution [27]. Our study therefore suggests that the sensitivity of particular genes to environmental changes could turn them into evolutionary hotspots by giving more substrate for selection.

## Results

### Temperature modulates the expression of the pigmentation gene *tan* in the posterior abdominal epidermis of females

**Fig 1:**
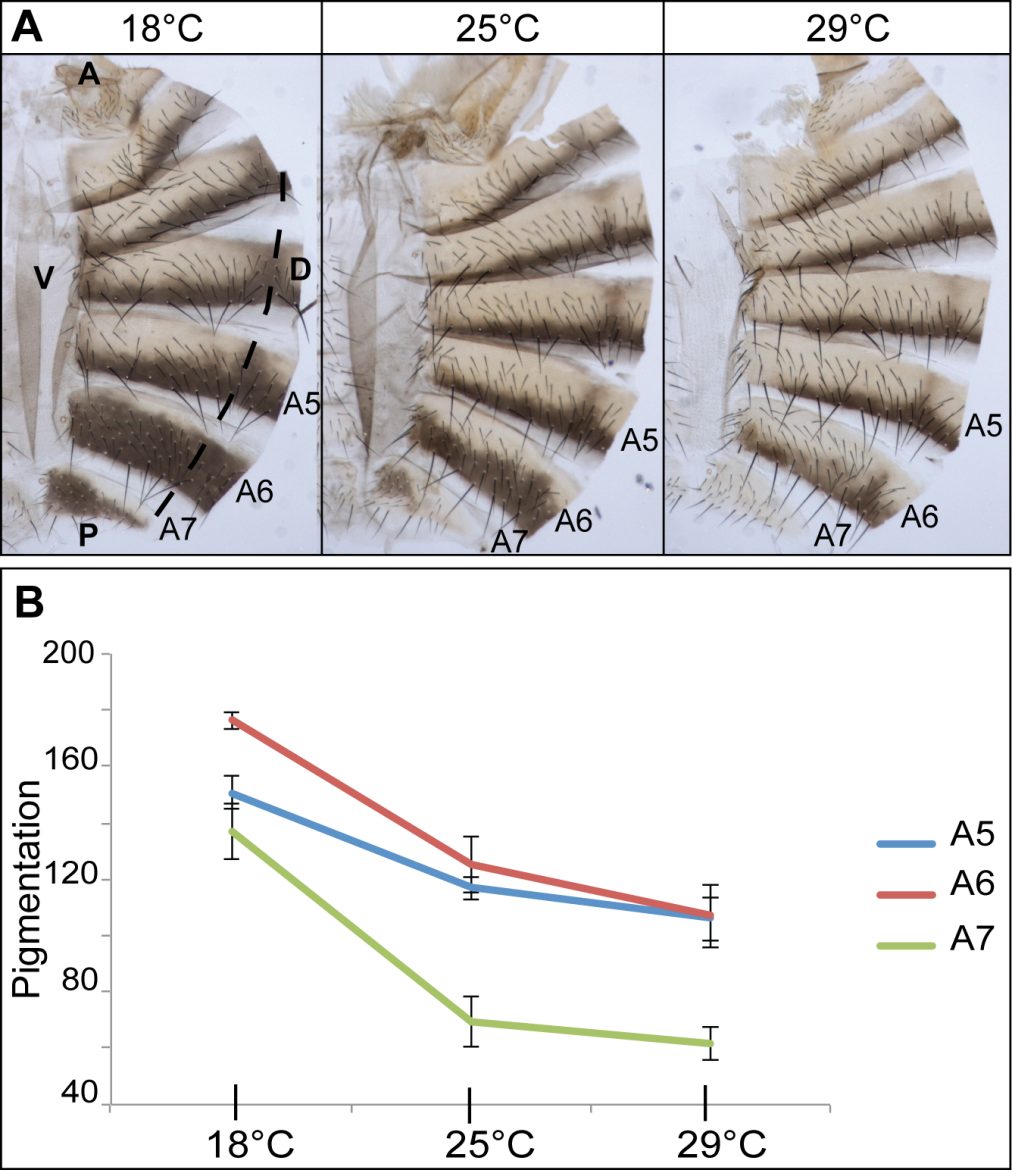
Plasticity of *Drosophila melanogaster* female abdominal pigmentation. (A) Abdominal cuticles of *w*^*1118*^ females grown at 18°C, 25°C or 29°C, showing strong thermal plasticity of pigmentation for abdominal tergites A5, A6 and A7. Cuticles were cut just beyond the dorsal midline (dotted line). Hemi-abdomens are shown. A: anterior, P: posterior, D: dorsal, V: ventral. (B) Reaction norms of pigmentation as a function of temperature in hemi-tergites A5, A6 and A7 of *w*^*1118*^ females (n = 10 *per* temperature), showing that A6 and A7 are the most plastic segments. Statistical tests to analyse the effect of temperature were ANOVA or Welch's ANOVA.

To focus on the effect of temperature, we quantified abdominal pigmentation in females from an inbred *w*^*1118*^ line, the wild-type stock commonly used in our laboratory for molecular experiments (Fig 1). As previously described for other *D*. *melanogaster* lines [14], flies raised at 18°C were darker than flies raised at 25°C or 29°C (Fig 1A). Female pigmentation plasticity was observed in the whole abdomen but was particularly pronounced in posterior abdominal segments A5, A6 and A7 (Fig 1B, A5: p<0.001; A6: p = 0.001; A7: p<0.001). Furthermore, statistical analyses revealed that temperature accounted for most of the variation of pigmentation (Eta-squared, A5: 0.91; A6: 0.93; A7: 0.95).

**Fig 2:**
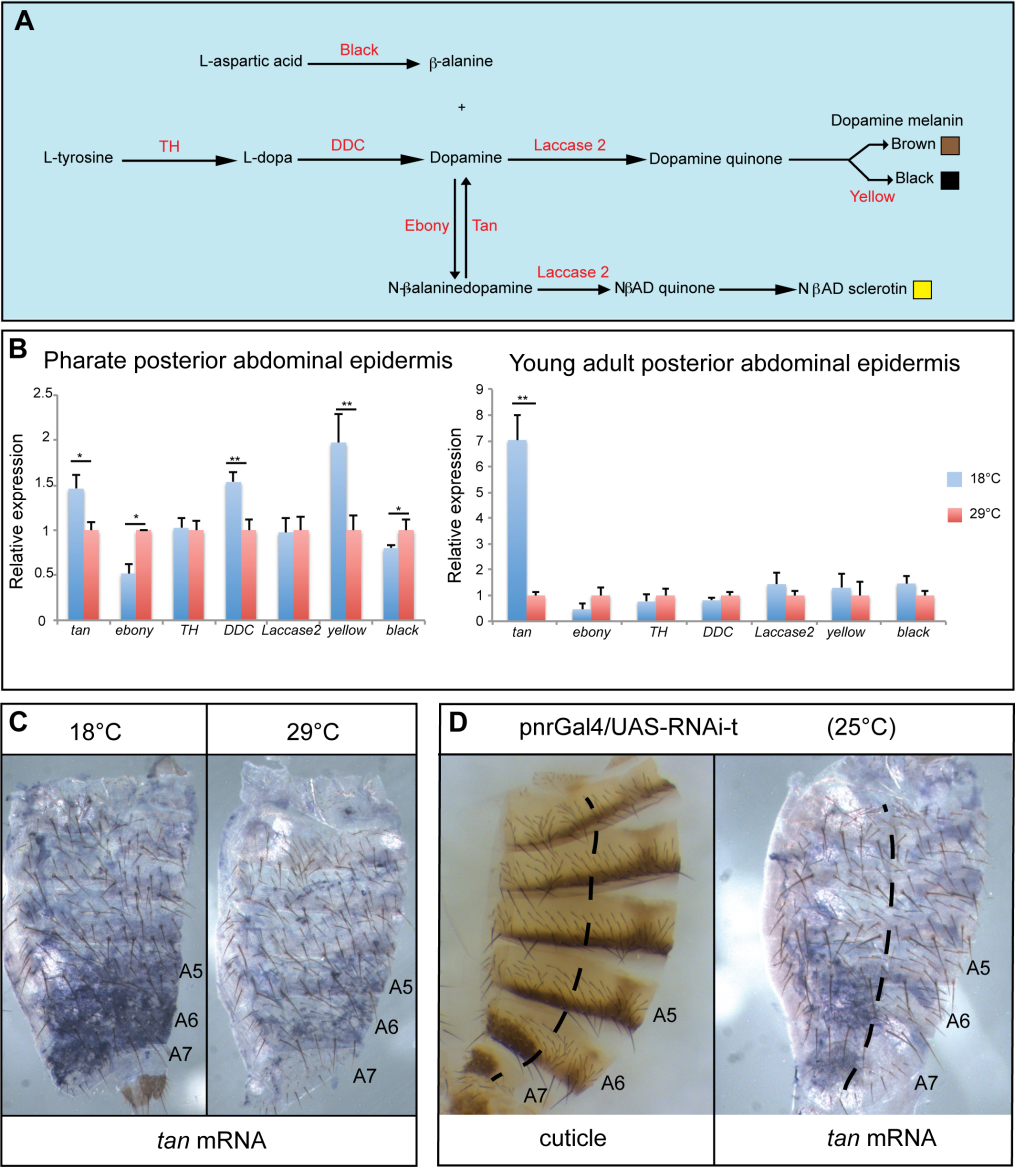
Temperature dramatically modulates the expression of the pigmentation gene *tan* in posterior abdominal epidermes of females. (A) Cuticle pigment synthesis pathway [28]. Enzymes are indicated in red. (B) Quantification of pigmentation gene expression in posterior abdomen epidermes (segments A5, A6 and A7) from female *w*^*1118*^ pharates (left) and young *w*^*1118*^ adult females (right) grown at 18°C or 29°C (pools of 50 epidermes for pharates and 30 epidermes for adults, n = 3, error bars: standard deviations; gene expression at 18°C has been normalized on gene expression at 29°C). The expression of *tan*, *ebony*, *DDC*, *yellow* and *black* is moderately modulated by temperature in pharates, whereas only *tan* is dramatically modulated in young adults (t-test: *: p<0.05; **: p<0.01). The expression of *Tyrosine Hydroxylase* (*TH*) and *Laccase 2* is modulated neither in pharates nor in adults. (C) Analysis of *tan* expression in abdominal epidermes from young *w*^*1118*^ adult females grown at 18°C or 29°C. Note that *tan* is more strongly expressed in the posterior abdominal epidermis at 18°C than at 29°C. (D) Adult cuticle (left) and *tan* expression in abdominal epidermis (right) from females in which *tan* was down-regulated using the *pnr-Gal4* driver and a *UAS-RNAi-t* transgene. The dashed line marks the limit between the *pnr* driver expression domain (a dorsal strip) and the lateral region used as an internal control. Note the loss of pigmentation (left panel) and the strong decrease in *tan* expression (right panel) in the dorsal region, showing specificity of *tan* antisense probe.

Cuticle pigmentation is a complex trait that involves the coordinated expression of many pigmentation enzyme coding genes, expressed from the second half of pupal life to the beginning of adulthood depending on the gene [28,29] (Fig 2A). To test whether the expression of these genes was modulated by temperature, we performed RT-qPCR experiments on epidermes of A5, A6 and A7 segments from *w*^*1118*^ females grown at 18°C or 29°C and collected at late pupal stage (pharates, Fig 2B left), or within two hours after eclosion, *i*.*e*. when cuticle tanning occurs (young adults, Fig 2B right). In pharates, the expression of *tan* (*t*), *ebony* (*e*), *Dopa Decarboxylase* (*DDC*), *yellow* (*y*) and *black* (*b*) was moderately modulated by temperature (less than 2 times). In young adults, among all genes tested, only *t* showed a significant modulation of expression by temperature. This modulation was very strong as *t* was expressed 7 times more at 18°C than at 29°C (Fig 2B, p<0.01). We therefore focused on *t* and we analysed its spatial expression by *in situ* hybridization in *D*. *melanogaster* female abdominal epidermis (line *w*^*1118*^) (Fig 2C and 2D). *t* was strongly expressed in the posterior abdomen of females grown at 18°C, as previously shown for *D*. *yakuba* females whose abdomen is darkly pigmented [30]. However, in *D*. melanogaster, *t* expression was strongly reduced at 29°C, which correlates with the lighter pigmentation of adult females. As *t* activity increases melanin production ([31] and Fig 2A), its changing expression with temperature might be directly linked to abdominal pigmentation plasticity of females.

### Temperature modulation of *tan* expression is essential for abdominal pigmentation plasticity in females

**Fig 3:**
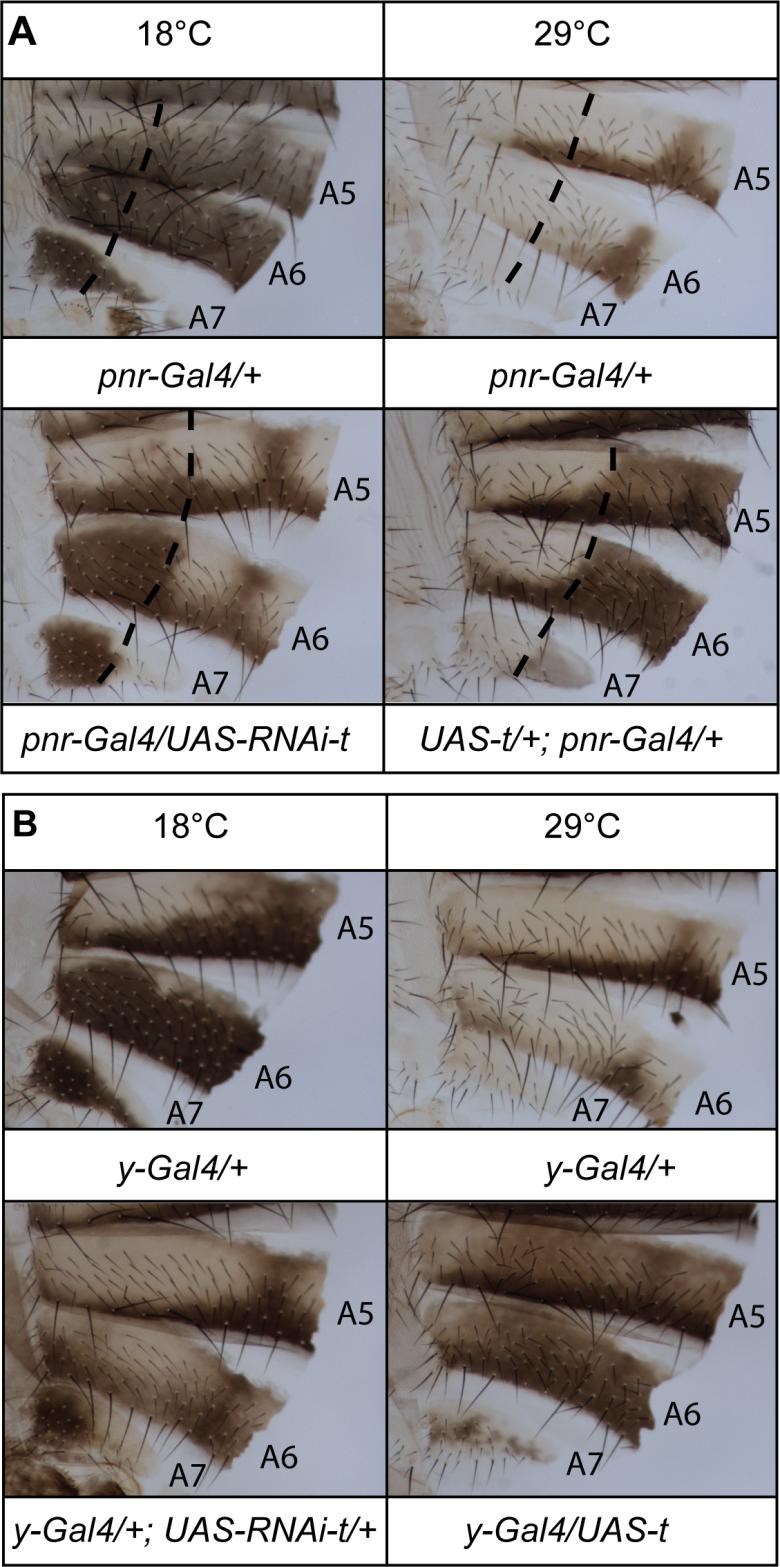
Modulation of *tan* expression is necessary and sufficient for female abdominal pigmentation plasticity. Genetic manipulation of *tan* with the *pnr-Gal4* (A) or the *y-Gal4* (B) driver shows that modulation of *tan* expression plays a major role in thermal plasticity of female abdominal pigmentation. Left (A and B): *tan* down-regulation at 18°C (*UAS-RNAi-t* transgene) is sufficient to reduce pigmentation. Right (A and B): *tan* over-expression at 29°C (*UAS-t* transgene) is sufficient to increase pigmentation. In (A), dashed lines mark left borders of the *pnr* driver expression domain.

If modulation of *t* expression by temperature were necessary and sufficient for thermal plasticity of female abdominal pigmentation, then manipulating *t* expression should counteract the effect of temperature. To test this hypothesis, we down-regulated or over-expressed *t* throughout development using the *pannier-Gal4* driver [32] *(pnr-Gal4)* combined with a *UAS-RNAi-t* ([33]) or a *UAS-t* ([31]) transgene (Fig 3A). As *pnr* is expressed only in the dorsal region of the body [32], the lateral regions serve as internal controls. *t* down-regulation at 18°C was sufficient to reduce pigmentation, which shows that high *t* expression at low temperature is required for dark pigmentation. Conversely, *t* over-expression at 29°C was sufficient to increase pigmentation, proving that at high temperature the lower level of *t* expression is limiting for melanin production. Similar results were obtained with *yellow-wb-Gal4* (*y-Gal4*), a driver expressed in wing and body epidermes at the late pupal stage (Fig 3B). These results show that modulation of *t* expression by temperature plays a major role in thermal plasticity of female abdominal pigmentation.

In the pigment synthesis pathway, *e* encodes the enzyme that synthesizes the substrate of Tan (Fig 2A). We thus wondered whether a functional *e* gene was required to observe the effect of *t* modulation on pigmentation. To test this, we manipulated *t* expression in an *e* loss-of-function mutant background (*e*^*1*^ allele). *t* mis-regulation had no phenotypic consequence on pigmentation in this background (S1 Fig), showing that *e* is epistatic over *t*. Hence, a functional *e* gene is required to observe the phenotypic effect of *t* expression modulation. This result again points towards *t* as the major effector of pigmentation thermal plasticity.

**Fig 4:**
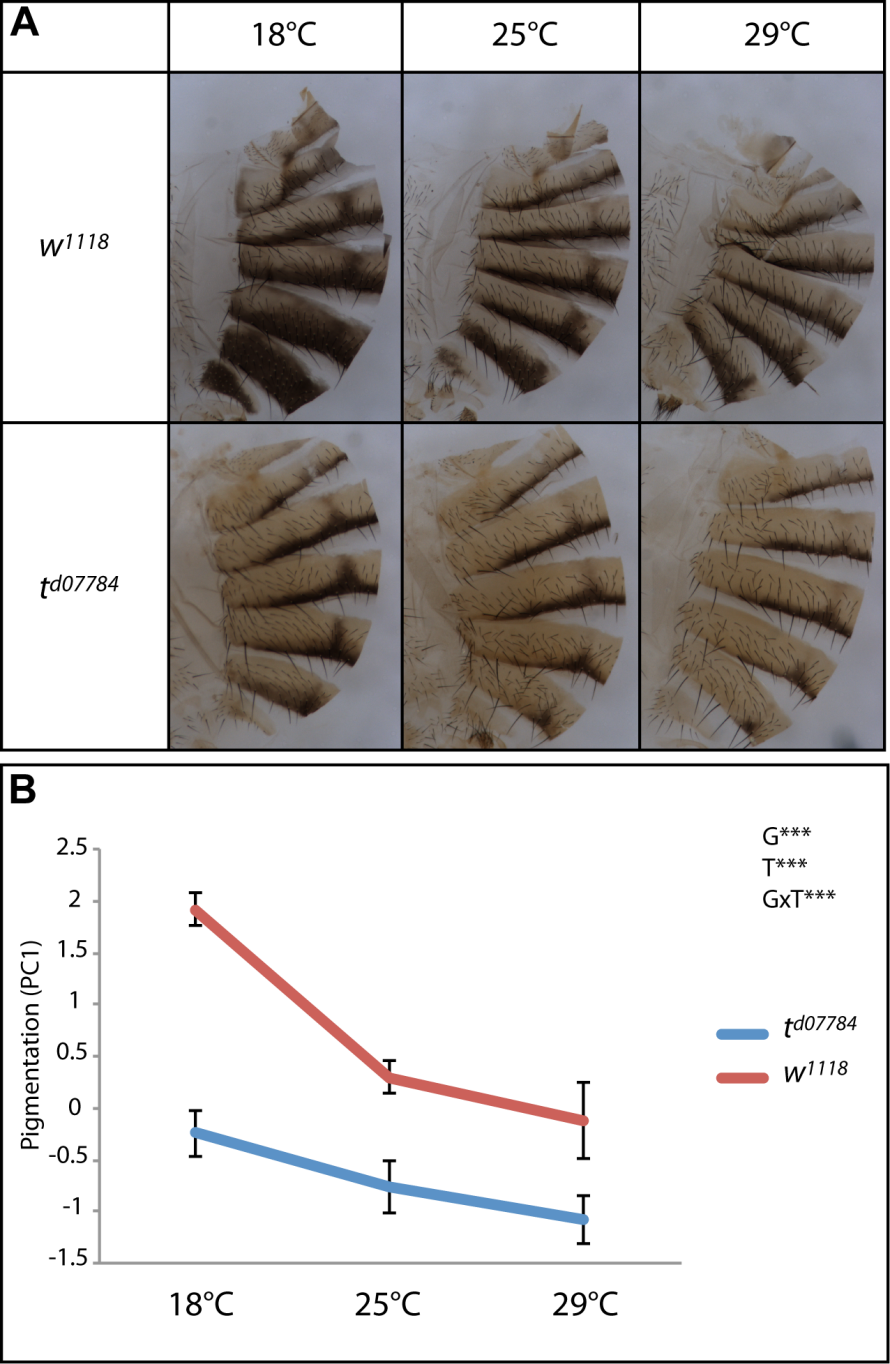
*tan* is involved in female abdominal pigmentation plasticity. (A) Cuticles of control (*w*^*1118*^) and *tan* mutant females (*t*^*d07784*^) grown at 18°C, 25°C and 29°C. (B) Reaction norms of the same genotypes (n = 10 per condition). The pigmentation value corresponds to the first component of a principal component analysis of pigmentation in segments A5, A6 and A7 that captures more than 95% of the total variance. There is a significant decrease in thermal plasticity of abdominal pigmentation in *tan* mutant females. Statistical test: two-way ANOVA. ***: p<0.001.

Involvement of a gene in thermal plasticity is quantified by the effect of the interaction between genotype and temperature. To further establish the role of *t* in thermal plasticity of female abdominal pigmentation, we compared the reaction norms [pigmentation = f(temperature)] of control flies and of *t* loss-of-function mutant flies (*t*^*d07784*^ allele) (Fig 4, S2 Fig). We observed a very strong effect of temperature (T, p<0.001; Eta-squared = 0.38) and of genotype (G, p<0.001; Eta-squared = 0.49) alone. As *t* is involved in abdominal pigmentation [31], this result was expected. In addition, the effect of the interaction between genotype and temperature was also very strong (GxT, p<0.001; Eta-squared = 0.08). Hence, *t*^*d07784*^ females are less plastic than wild type females, thus corroborating the role of *t* in thermal plasticity of abdominal pigmentation.

### Regulation of *tan* expression by temperature implicates an abdominal epidermis enhancer

**Fig 5:**
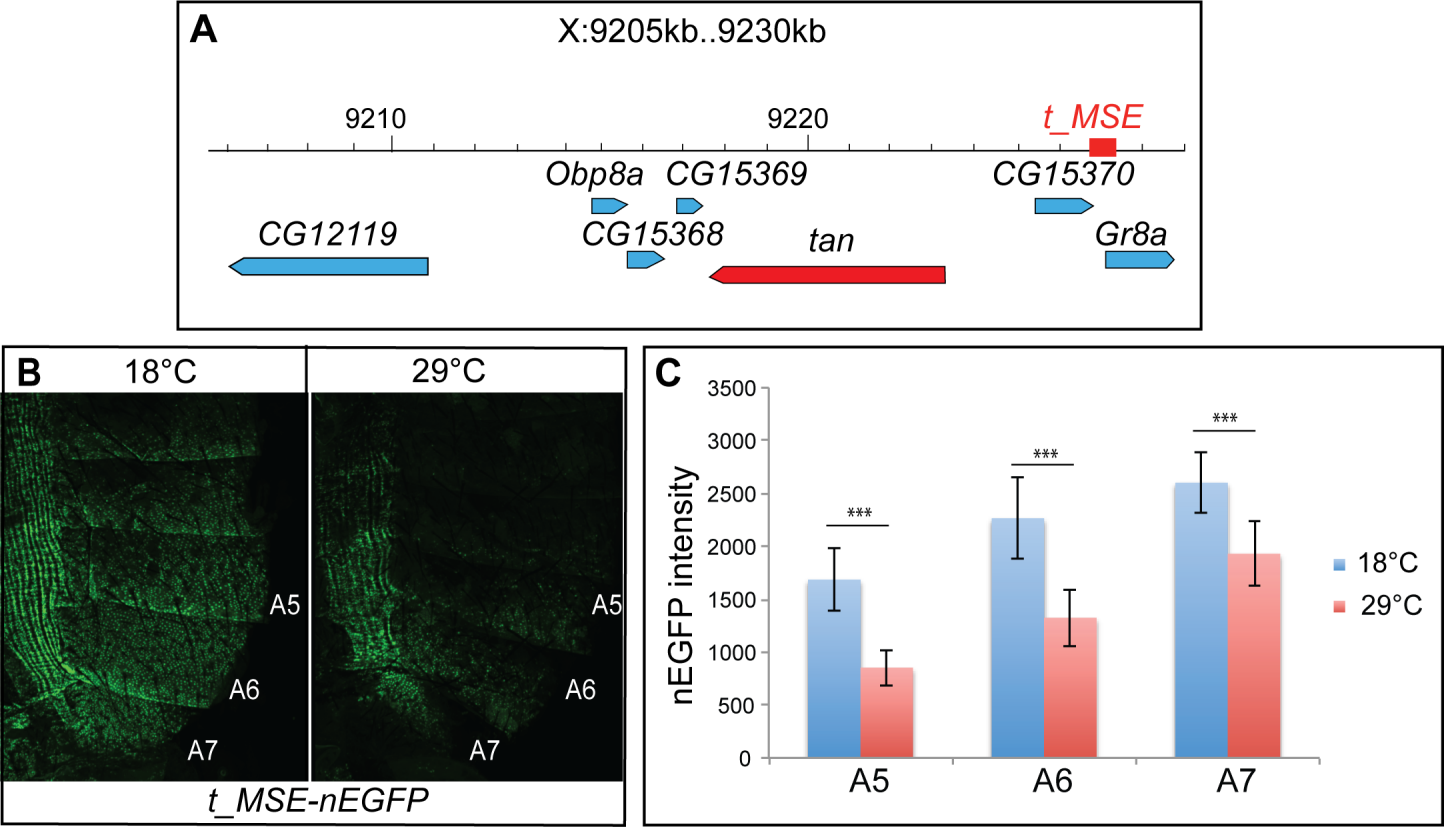
Temperature regulates the activity of an abdominal epidermis enhancer of *tan*, *t_MSE*. (A) *tan* genomic region (after Flybase, http://flybase.org/) showing the location of *t_MSE* between the genes *CG15370* and *Gr8a*. (B, C) The activity of *t_MSE* (*t_MSE-nEGFP* reporter transgene) in abdominal epidermes of young adult females is modulated by temperature. (B) nEGFP fluorescence in abdominal epidermes at 18°C and 29°C. Fluorescence on the left part of the tissue corresponds to the pleura. (C) Quantification of nEGFP fluorescence in A5, A6 and A7 hemi-tergites at 18° and 29°C (n = 10 *per* temperature). nEGFP intensity is higher at 18°C than at 29°C (t-test; ***: p<0.001).

The effect of temperature on *t* expression could be mediated by its *cis-*regulatory sequences. An enhancer essential for driving *t* expression in the epidermis of abdominal segments A5 and A6 in males, *t_MSE*, was previously mapped upstream of *t*, between the genes *CG15370* and *Gr8a* [17] (Fig 5A). We analysed the activity of a *t_MSE-nEGFP* reporter transgene [17] in young females grown at 18°C and 29°C. Quantification of nEGFP in segments A5, A6 and A7 showed that this enhancer was also active in female abdominal epidermes. Furthermore, its activity was modulated by temperature, as nEGFP was between 1.3 and 2 times more expressed at 18°C than at 29°C, depending on the segment (Fig 5B and 5C, p<0.001). When using an *ebony-nEGFP* transgene in which nEGFP is under control of the regulatory sequences of *ebony* [34], a pigmentation gene not modulated by temperature in the posterior abdominal epidermis of young females (Fig 2B and S3A Fig), we observed no higher nEGFP expression at 18°C as compared to 29°C (S3B and S3C Fig). This indicates that transcription of *nEGFP* and not stability of the nEGFP protein was responsible for the effect observed with the *t_MSE-nEGFP* transgene. Interestingly, the fold change observed with the *t_MSE-nEGFP* transgene between 18°C and 29°C was lower than that of *t* expression (Fig 2B). This could be due to the genetic background. Alternatively, additional regulatory sequences of *t* may be important to mediate the effect of temperature. In conclusion, these results show that the effect of temperature on *t* expression is mediated, at least partly, by *t_MSE*.

### Temperature affects chromatin configuration in *tan* region

**Fig 6:**
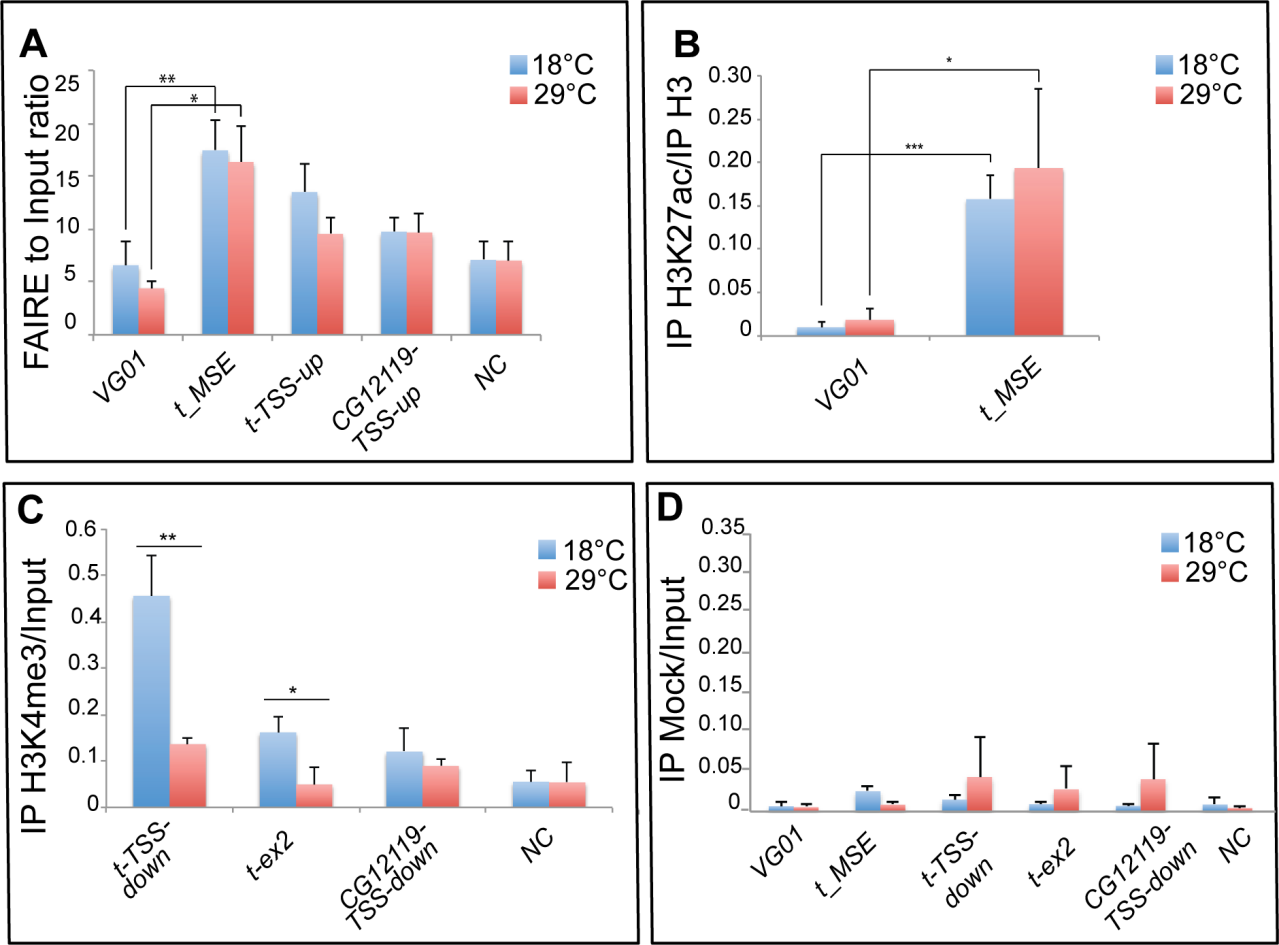
Chromatin configuration at the *tan* locus in abdominal epidermes of females grown at 18°C or 29°C. (A) *t_MSE* is less compact than the silent *VG01* enhancer at 18°C and 29°C, but showed no difference in compaction between 18°C and 29°C. By contrast, chromatin upstream the TSS of *tan* (*t-TSS*-*up*) tends to be less compact at 18°C than at 29°C. *CG12119-TSS-up*: region upstream of the TSS of *CG12119*. *NC*: untranscribed region between *tan* and *CG12119*. (B) H3K27ac is significantly enriched on *t_MSE* as compared to *VG01*, but is not modulated by temperature. H3K27ac IP signal was normalized to panH3 IP signal as the amount of H3 was higher for *VG01* than for *t_MSE* (S5A Fig). When normalized to input, a similar pattern was observed (S5B Fig). (C) H3K4me3 is modulated by temperature downstream of the TSS of *tan* (*t-TSS-down*) and on *tan* exon 2 (*t-ex2*), but not downstream the TSS of *CG12119* (*CG12119-TSS-down*) or in an untranscribed control region (*NC*). H3K4me3 IP signal was normalized to input signal because the amounts of H3 were similar for all regions tested (S5A Fig). When normalized to panH3 IP signal, a similar pattern was obtained (S5C Fig). (D) Mock IP signal normalized to Input signal for *VG01*, *t_MSE*, *t-TSS-down*, *t-ex2*, *CG12119-TSS-down* and *NC*. Graphs in A, B, C, D represent the mean of three independent experiments, the error bars correspond to standard deviations. Statistical analysis: t-test; *: p<0.05; **: p<0.01; ***: p<0.001.

Modulation of *t_MSE* activity by temperature prompted us to analyse the chromatin structure of this enhancer in epidermes of female abdominal segments A5, A6 and A7 at 18°C and 29°C (Fig 6). As nucleosome depletion characterizes active regulatory chromatin regions [35,36], we performed Formaldehyde Assisted Isolation of Regulatory Elements (FAIRE)-qPCR experiments, a methodology allowing detection of open chromatin [37,38]. FAIRE experiments have previously shown that the *VG01* enhancer of *vestigial (vg)*, which recapitulates *vg* expression in wing and haltere imaginal discs, was specifically open in these tissues, but not in leg imaginal discs where *vg* is not expressed [38]. As *vg* is not expressed in the abdominal epidermis either (S4A Fig), we used *VG01* as a negative control. FAIRE signal was significantly higher on *t_MSE*, showing that *t_MSE* was less compact than *VG01* at 18°C and 29°C (18°C: p<0.01; 29°C: p<0.05) (Fig 6A). However, compaction of *t_MSE* was similar at 18°C and 29°C. Similar conclusions were drawn from analysis of total histone 3 (panH3) enrichment by chromatin immunoprecipitation experiments (ChIP-qPCR), which showed a higher nucleosome concentration on the *VG01* enhancer than on *t_MSE*, but no difference between 18°C and 29°C for both enhancers (S5A Fig). We then analysed the enrichment of *t_MSE* in H3K27ac, a histone mark characteristic of active enhancers [39]. *t_MSE* was enriched in H3K27ac compared to *VG01* enhancer at both 18°C (p<0.001) and 29°C (p<0.05). However, we detected no significant H3K27ac enrichment on *t_MSE* at 18°C compared to 29°C (Fig 6B and S5B Fig). This result indicates that *t_MSE* is active at 18°C and at 29°C. Furthermore, temperature affects neither the compaction of *t_MSE* nor the apposition of H3K27ac. However, other histone marks on *t_MSE* might be modulated by temperature. Alternatively, the effect of temperature on chromatin structure could target another region of *t*, for example its promoter. We thus studied chromatin compaction and the H3K4me3 active mark at the *t* promoter. The 500 base pair region upstream of the transcription start site (TSS) of active genes, which includes the promoter, is known to be depleted in nucleosomes [40]. FAIRE-qPCR experiments showed that chromatin upstream the *t* TSS (*t-TSS*-*up*, -253 to -151 bp) tended to be less compact at 18°C than at 29°C (Fig 6A, p = 0.087), which correlated with the higher expression of *t* at 18°C compared to 29°C. No such difference was observed for *CG12119* (Fig 6A, *CG12119-TSS-up*, -266 to -200 bp), a gene nearby *t* (Fig 5A) that was expressed at the same level at 18°C and 29°C (S4B Fig), or for an untranscribed region between *CG12119* and *t* (Fig 6A, *NC*). Highly transcribed genes are enriched in H3K4me3, with a maximum of enrichment 50–750 bp downstream of the TSS [41]. We found that H3K4me3 was strongly enriched at 18°C as compared to 29°C both downstream of *t* TSS (Fig 6C, *t-TSS down*, 193 to 288 bp, p<0.01; S5C Fig) and on *t* exon 2 (Fig 6C, *t-ex2*, p<0.05; S5C Fig). Such a difference between 18°C and 29°C, which correlates with higher *t* expression at 18°C, was not observed for *CG12119-TSS-down* (204 to 256 bp) or for *NC* (Fig 6C and S5C Fig).

In conclusion, our results show that temperature modulates chromatin compaction and H3K4me3 enrichment on the *t* promoter in the posterior abdominal epidermis of females.

### *trithorax* participates in *tan* regulation and thermal plasticity of pigmentation

As temperature modulates deposition of the H3K4me3 active mark on *t*, we addressed the role of genes involved in H3K4 methylation in pigmentation and its plasticity. In *D*. *melanogaster*, H3K4 mono-, di- and tri- methylations are catalysed by three complexes of the COMPASS family called Trithorax (Trx), Trithorax-related (Trr) and Set1. These complexes are characterised by their histone methyl-transferase subunit encoded by the genes *trx*, *trr* and *Set1*, respectively [42]. The histone methyl-transferase Trx was also purified previously from another complex, TAC1 [43]. Whereas Trr is involved in H3K4 mono-methylation [44], Set1 is responsible for the bulk of H3K4 di- and tri-methylation [45]. Independent studies indicate a role for Trx in H3K4 mono- and tri-methylation [45–47].

**Fig 7:**
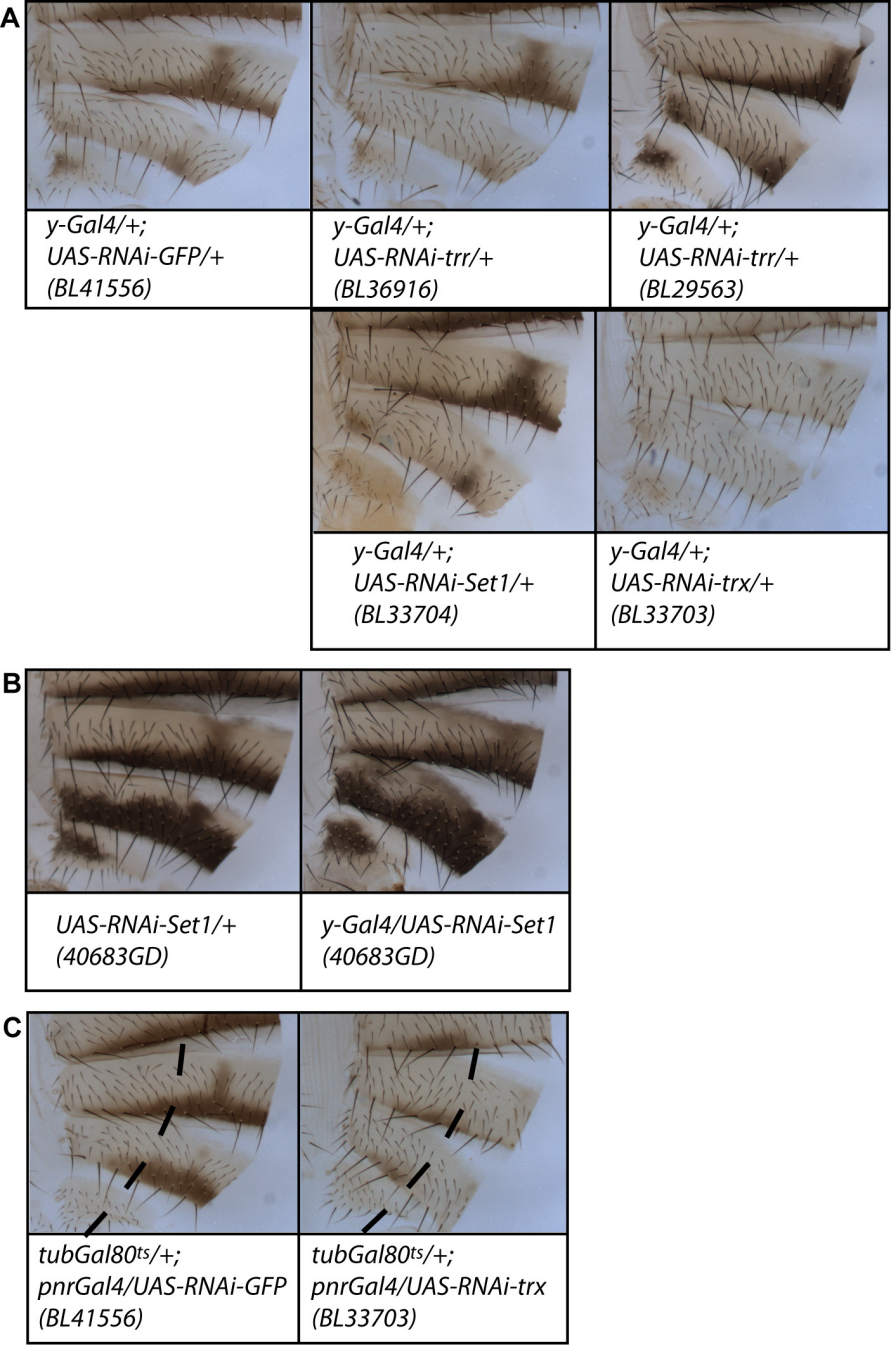
*trx* is involved in female abdominal pigmentation, whereas *trr* and *Set1* are not. (A, B) When using the *y-Gal4* driver and *UAS-RNAi* transgenes at 25°C, *trx* down-regulation induces abdominal depigmentation, whereas *trr* or *Set1* down-regulation does not. In A, the effect of the RNAi transgenes against *trr*, *Set1* or *trx* (*VALIUM RNAi* lines) was compared to that of an RNAi transgene against *GFP* inserted at the same site in the same genetic background. In B, the RNAi line against *Set1* (VDRC line) driven by *y-Gal4* was compared with females heterozygous for the transgene. (C) *trx* down-regulation during late pupal stage (*pnr-Gal4* driver in combination with *tub-Gal80*^*ts*^ transgene) induced abdominal depigmentation. Dashed lines mark left borders of the *pnr* driver expression domain. The *UAS-RNAi-GFP* transgene is used as a negative control.

We first down-regulated *trx*, *trr* or *Set1* using *UAS-RNAi* transgenes and the late pupal driver *y-Gal4* to analyse their implication in abdominal pigmentation (Fig 7A and 7B). Down-regulation of *trr* and *Set1* using two different RNAi lines for each gene induced no changes in pigmentation. By contrast, *trx* down-regulation induced strong depigmentation of all abdominal segments. A similar phenotype was obtained by inducing *trx* down-regulation at late-pupal life with the *pnr-Gal4* driver combined with *Gal80*^*ts*^ (Fig 7C). These results show that Trx, but neither Trr nor Set1, participates in the late steps of female abdominal pigmentation establishment.

**Fig 8:**
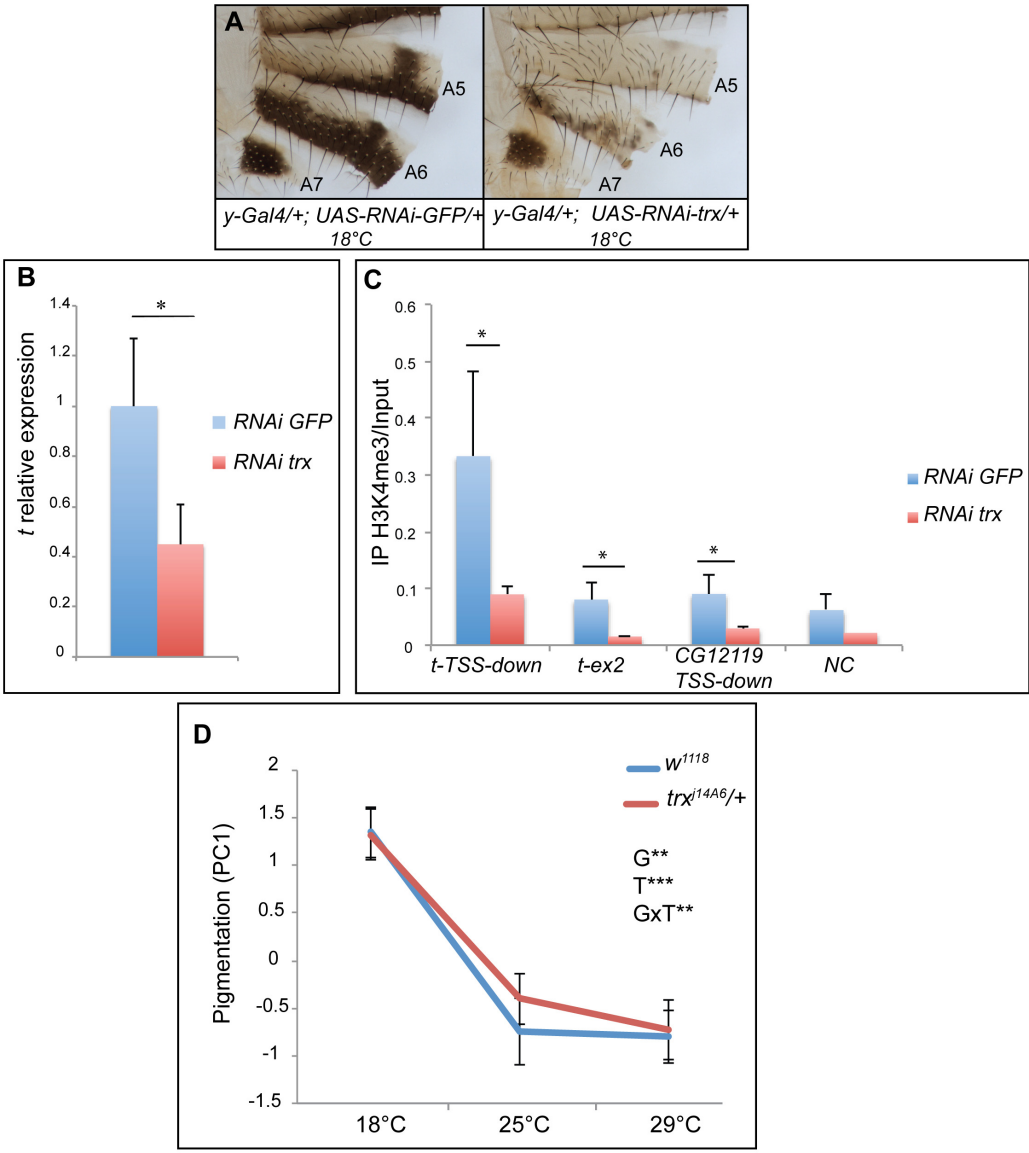
*trx* regulates *tan* expression and is involved in female abdominal pigmentation and its plasticity. (A) Down-regulation of *trx* at 18°C in the abdominal epidermis (*y-Gal4* driver, *UAS-RNAi*-*trx* transgene) induced strong depigmentation. (B) Quantification of *tan* expression in posterior abdominal epidermes (segments A5, A6 and A7) from young *y-Gal4*>*UAS-RNAi*-*trx* and *y-Gal4>UAS-RNAi-GFP* females grown at 18°C (pools of 30 epidermes, n = 3, error bars: standard deviations; *t* expression in *y-Gal4*>*UAS-RNAi*-*trx* females was normalized on *t* expression in *y-Gal4>UAS-RNAi-GFP* females). *trx* down-regulation induced a decrease in *tan* expression (t-test; *: p<0.05). (C) Analysis by chromatin immuno-precipitation of the H3K4me3 mark in abdominal epidermes of young *y-Gal4*>*UAS-RNAi*-*trx* and *y-Gal4>UAS-RNAi-GFP* females grown at 18°C. *trx* is required for the high level of H3K4me3 downstream the TSS of *tan* (*t-TSS-down*), on *tan* exon 2 (*t-ex2*) and downstream the TSS of *CG12119* (*CG12119-TSS-down*), a gene near *tan* expressed in the abdominal epidermis. *NC*: untranscribed region between *tan* and *CG12119*. The graph represents the mean of three independent experiments, the error bars correspond to standard deviations. (D) Pigmentation reaction norms (n = 30 *per* genotype and *per* temperature) showed that thermal plasticity is significantly different between *w*^*1118*^ and *trx*^*j14A6*^*/+* females. Statistical test: two-way ANOVA. **: p<0.01; ***: p<0.001. T: effect of temperature; G: effect of genotype; GxT: effect of the interaction between genotype and temperature. Pigmentation corresponds to the first principal component (PC1) extracted from pigmentation in segments A5, A6 and A7 that captures more than 95% of total variance.

As the level of H3K4me3 on the *t* promoter was modulated by temperature, we wondered whether Trx participates in *t* regulation. We thus quantified *t* expression in abdominal epidermes of *y-Gal4>UAS-RNAi-trx* females raised at 18°C, a temperature at which loss of pigmentation induced by *trx* down-regulation was very strong (Fig 8A). *trx* down-regulation induced a significant decrease in *t* expression (Fig 8B, 2.1 fold down, p<0.05), showing that *trx* is required for the strong expression of *t* in abdominal epidermes at 18°C. In addition, down-regulation of *trx* in abdominal epidermes significantly reduced H3K4me3 on the *t* promoter (Fig 8C, 3.7 fold down, p<0.05), which suggests that Trx participates in H3K4me3 deposition on *t*.

Interestingly, *trx RNAi* females exhibited stronger loss of melanin than *t*^*d07784*^ mutants suggesting that Trx controls the expression of other pigmentation genes. Therefore, we analysed the expression of pigmentation genes in the abdominal epidermis of *y-Gal4>UAS-RNAi-trx* females raised at 18°C (S6 Fig). In addition to *t*, *TH*, *DDC* and *b* were down-regulated showing that Trx also participates in their regulation.

We then investigated the involvement of *trx* in thermal plasticity of pigmentation. As we could not use a *trx UAS-RNAi* transgene since the *UAS/Gal4* system is temperature-sensitive, we established the pigmentation reaction norms of *trx*^*j14A6*^ heterozygous mutant females (Fig 8D and S7 Fig). The effect of this allele on pigmentation was not as strong as the one of the *trx UAS-RNAi* transgene, probably because only heterozygous females could be studied (the *trx*^*j14A6*^ allele is lethal homozygous). Nevertheless, the interaction between genotype and temperature was highly significant (Fig 8D, GxT, p<0.01), indicating that *trx* is involved in thermal plasticity of pigmentation.

## Discussion

We show here for the first time that thermal plasticity of female abdominal pigmentation in *D*. *melanogaster* involves strong modulation of the expression of the pigmentation gene *t*. Furthermore, our results demonstrate that this modulation plays a major role in female abdominal pigmentation plasticity. Interestingly, a previous study analysing thermal plasticity of gene expression in the whole body of three days old *D*. *melanogaster* females showed that *t* expression diminishes when temperature increases [48]. However, as the abdominal pigmentation pattern is already established at this stage, it is likely that, in these experiments, other tissues contribute to the variation of *t* expression. As *t* is expressed in photoreceptors and plays a role in vision [31], it would be interesting to test whether its expression varies with temperature in adult eyes.

In young adults, *t* is the only pigmentation gene among those tested which is significantly modulated by temperature. However, we observed a trend towards a weaker *e* expression at 18°C than at 29°C, although not statistically significant (Fig 2B, p = 0.06). In pharates, several pigmentation genes, including *t* and *e*, are moderately modulated by temperature. In addition, we observed a weaker expression of *e-nEGFP* in A6 and A7 at 18°C than at 29°C (S3 Fig). These findings agree with our previous data showing the qualitative analysis of *e* expression at different temperatures using an *e-lacZ* transgene [25]. In this previous publication we showed that *e* mutants remain dark at all temperatures and concluded that "a functional *e* gene is required for the plasticity of pigmentation". Our present data complete this conclusion. Indeed, we show here that *e* is epistatic over *t*. This explains why *e* mutants lose abdominal pigmentation plasticity, as a functional *e* gene is required to observe plasticity induced by modulation of *t* expression.

Furthermore, our data show that the expression of *e*, *DDC*, *y* and *b* is modulated by temperature in pharates. This could explain the residual pigmentation plasticity observed in *t* mutants.

Lastly, spatial analysis of *e* expression by *in situ* hybridization reveals a stronger expression at 29°C than at 18°C in anterior abdominal segments. This observation suggests that the reduced but observable plasticity of these anterior segments might be due to *e* temperature sensitive expression.

The effect of temperature on *t* expression is mediated, at least partly, by the *t_MSE* enhancer. Thus, this enhancer may have particular properties making it temperature sensitive. Indeed, recent data showed that the number of redundant binding sites for a particular transcription factor in an enhancer could influence its temperature sensitivity [49]. Another, non-exclusive, explanation could be that temperature affects the expression or the activity of regulatory factors upstream of *t*. We detected no chromatin modification of *t_MSE* at different temperatures, possibly because this enhancer is active, although at different levels, at the temperatures we tested. The level of H3K27ac could therefore be saturated and the chromatin on *t_MSE* decompacted at both temperatures. By contrast, the effect of temperature on *t* expression is correlated with the modulation of H3K4me3 deposition on the *t* promoter. As this histone mark correlates with active transcription [50], the strong accumulation of *t* transcripts at 18°C is more likely caused by a transcriptional response to temperature than by modulation of a post-transcriptional mechanism that would stabilize them. Interestingly, deposition of H3K4me3 can also be modulated by environmental conditions such as diet in mouse liver [51], drought stress in plants [52,53] or chemical stress in yeast [54]. This histone mark emerges therefore as a general mediator of environmental impact on the genome.

We show that the H3K4 methyl-transferase Trx is involved in *t* regulation, but also in the regulation of other pigmentation genes. As the level of H3K4me3 on the *t* promoter decreases when *trx* is inactivated, it is tempting to speculate that Trx directly regulates *t*. However, Trx might also indirectly control *t* expression through the regulation of genes upstream *t*. Furthermore, as Trx has no intrinsic DNA binding activity, its recruitment on *t* or on upstream regulators must depend on specific transcription factors. Thus, it would be interesting to identify the upstream regulators of *t* controlled by Trx as well as the transcription factors recruiting Trx on *t* or on its upstream regulators.

Trx also participates in the thermal plasticity of female abdominal pigmentation. This confers to Trx a very specific role as compared to other H3K4 methyl-transferases. Indeed, Set1 has been described as the main H3K4 di- and tri- methyl-transferase during *Drosophila* development [45]. However, our results demonstrate for the first time that Trx is involved in the thermal plasticity of female abdominal pigmentation.

Modulation of pigmentation by environmental conditions is observed in many insects [55,56]. Interestingly, *t* expression is strongly modulated by environmental conditions in the developing wings of *Junonia coenia*, a butterfly with contrasting seasonal morphs [57]. The involvement of *t* in pigmentation plasticity might therefore be widespread in insects.

Several studies have also linked *t* to pigmentation variation within or between *Drosophila* species. Modulation of *t* expression through modification of *t cis*-regulatory sequences has been implicated in evolution of abdominal pigmentation between species [17,19,26]. Remarkably, in *D*. *santomea*, independent mutations in *t_MSE* have generated three distinct loss-of-function alleles involved in the reduced pigmentation of this species [17]. Furthermore, SNPs associated with variation of abdominal pigmentation in *D*. *melanogaster* females have been identified in *t_MSE* [20]. Interestingly, abdominal pigmentation dimorphism in female *Drosophila erecta* was recently shown to be caused by sequence variation in *t_MSE* maintained by balancing selection [58]. The recurrent implication of *t* in pigmentation evolution has led to list this gene among hotspots of evolution [27]. In other organisms, genes sensitive to environment and involved in phenotypic plasticity are also responsible for differences within or between species. For example, in *Brassicaceae*, the *reduced complexity* locus *(RCO)* that participates in leaf margin dissection is modulated by temperature and has been repeatedly involved in leaf shape evolution through *cis-*regulatory sequence variation or gene loss [59]. Therefore, sensitivity of particular genes to environmental conditions might turn them into evolutionary hotspots. Indeed, this broadens the range of phenotypes produced by a particular allele, providing more substrate for natural selection.

## Materials and Methods

### Fly stocks

We used a *w*^*1118*^ inbred line as wild-type. The *UAS-t* line was a gift from Dr. Nicolas Gompel, whereas *t_MSE-nEGFP* was from Dr. Sean Carroll's lab. The *ebony-nEGFP* line (*ebony-(ABC+intron)-nEGFP*) was from Dr. Mark Rebeiz. The *UAS-RNAi-t* (GD18124) and *UAS-RNAi-Set1* (GD40683) lines were from the VDRC Stock Center. The *pnr-Gal4* (BL3039), *y-Gal4* (BL44267), *P(XP)t*^*d07784*^ (BL19282), *e*^*1*^ (BL1658), *trx*^*j14A6*^ (BL12137), as well as the *VALIUM UAS-RNAi* lines (Transgenic RNAi Project at Harvard Medical School) against *trx* (BL33703), *trr* (BL29563 and BL36916), *Set1* (BL33704) and *GFP* (BL41556) were from the Bloomington Stock Center. The homozygous lethal *trx*^*j14A6*^ allele that corresponds to an insertion of a *w+ P* transposon was used in this study. This allowed us to introgress this allele in the *w*^*1118*^ background (ten generations), so that the mutation is in the same genetic background as the control. Complementation test with a well characterized *trx* loss-of function allele (*trx*^*E2*^ [60]) indicated that *trx* ^*j14A6*^ is a genuine loss-of-function allele of *trx*. To control the expression of RNAi transgenes during development, we combined the *pnr-Gal4* driver with the *tub-Gal80*^*ts*^ transgene from the Bloomington Stock Center (BL7019). Gal80 inactivation was performed by shifting the progeny at late pupal stage from 18°C to 29°C. We tested that all lines allowing *trx*, *trr* or *Set1* down-regulation induced lethality with the ubiquitous *daughtherless-Gal4* (*da-Gal4*) driver. Efficiencies of BL29563 (*UAS-RNAi-trr*) and GD40683 (*UAS-RNAi-Set1*) were previously published [42,45]. For the *UAS-RNAi-trx* line (BL33703), quantification of *trx* expression level in *da-Gal4>UAS-RNAi-trx* embryos showed a 1.5 fold down regulation as compared to control embryos, thus proving its efficiency.

### Cuticle preparations

Adult females between 3 and 5 days old were stored for 10 days in ethanol 75% before dissection. Abdominal cuticles were cut just beyond the dorsal midline, which was therefore entirely included in each preparation. After dissection, cuticles were dehydrated 5 minutes in ethanol 100% and mounted in Euparal (Roth). For nEGFP observations, abdomens were dissected in PBS, fixed 20 minutes in 3.7% paraformaldehyde in PBS, washed twice 10 minutes in PBS and mounted in Mowiol.

### *In situ* hybridizations

Fragments of cDNAs from *t* (611 bp) and *e* (639 bp) were amplified by PCR (primer sequences are listed in S1 Table) and cloned by Topo-Cloning and LR-Recombination (Gateway) in *pBlueScript* vector (Invitrogen). Sense and antisense DIG-labelled RNA probes were synthesized using the appropriate RNA polymerase. *In situ* hybridizations were performed according to the Carroll's lab protocol (http://carroll.molbio.wisc.edu). Specificity of the antisense probe was assessed by comparison with signal from the sense probe. For *t*, we also performed *in situ* hybridization with the *t* antisense probe on *UAS-RNAi-t/pnrGal4* females and observed a strong decrease of the signal in the *pnr* domain (Fig 2D).

### Image acquisitions and quantifications

Adult cuticles and abdominal *in situ* hybridizations were imaged with a binocular equipped with a Leica DC480 digital camera using the Leica IM50 Image Manager software. They were imaged using identical settings and an annular lamp to ensure homogeneous lighting. To quantify pigmentation, each entire hemi-segment was circled by hand. For A5 and A6, the melanic line at the dorsal limit of each hemi-segment (*i*.*e* the dorsal midline) separates the two hemi-segments. Cuticle pigmentation in hemi-tergites A5, A6 or A7 was measured as mean grey value using ImageJ. This value was subtracted from 255 to get a final pigmentation value comprised between 0 (white) and 255 (black).

Abdominal epidermes of *t_MSE-nEGFP* and *ebony-nEGFP* females were imaged using a Macro-Apotome (Zeiss). nEGFP intensity was measured in hemi-tergites A5, A6 or A7 using ImageJ in Maximum Intensity projections of 40 picture stacks.

### RT-qPCR experiments

RNA was extracted from pools of dissected female posterior abdominal epidermes (A5, A6 and A7) with the RNAeasy Mini kit (Qiagen) (50 abdominal epidermes for pharates, 30 for young adults). We could not use developmental time to stage pharates as it is temperature sensitive. We therefore used morphological markers (wing colour, abdominal bristles, localisation of the meconium in anterior abdomen) to collect pharates grown at 18°C or 29°C at a similar developmental stage. This stage corresponds to the stage P12(i) described by Bainbridge and Bownes [61]. For each experiment three independent replicates were analysed for each genotype and each temperature except for S6 Fig (two replicates). After treatment of RNA with Turbo DNAse (Ambion), cDNA were synthesized with the SuperScript II Reverse transcriptase kit (Invitrogen) using random primers. RT-qPCR experiments were carried out in a CFX96 system (Biorad) using SsoFast EvaGreen Supermix (Biorad). Expression levels were quantified with the Pfaffl method [62]. The geometric mean of two reference genes (Fig 2B and S4 Fig: *rp49* and *Act5C*; Fig 8B: *rp49* and *eIF2*,) was used for normalization [63]. Primers used are listed in S1 Table.

### Chromatin immunoprecipitation experiments

Chromatin immunoprecipitation (ChIP) experiments were performed as previously described [64] with minor modifications. For each experiment, 50 posterior abdominal epidermes (A5, A6 and A7) of females between 0 and 2h after hatching and 3μg of antibody were used. Results present the mean of three independent experiments for each antibody. Tissue disruption was performed before cell lysis using the FastPrep technology (MP Biomedicals, Lysis matrix D, 20 seconds at 4m/s). Chromatin sonication was performed in a Bioruptor sonifier (Diagenode) (16 cycles of 30'' ON, 30'' OFF, High power). Input and immunoprecipitated DNA were purified with the Ipure kit (Diagenode) in 70μl of water and 4μl were used *per* qPCR reaction. qPCR experiments were carried out in a CFX96 system (Biorad) using SsoFast EvaGreen Supermix (Biorad). Primers used are listed in S1 Table. Data were normalized against input chromatin or panH3 ChIP. Antibodies used were anti-H3K4me3 (C15410003, Diagenode), anti-H3K27ac (C15410174, Diagenode), anti-panH3 (C15310135, Diagenode). Rabbit IgGs (Diagenode) were used as negative control (Mock).

### FAIRE experiments

75 posterior abdominal epidermes (A5, A6 and A7) of females between 0 and 2h after hatching were used for each FAIRE experiment. Fixation and lysis protocols were similar to those used for ChIP except that fixation was performed for 5 minutes at room temperature in PBS-1% paraformaldehyde with gentle shaking. Chromatin sonication was performed in 300μl in a Bioruptor sonifier (Diagenode) with 8 cycles of 30'' ON, 30'' OFF, High power, allowing to obtain chromatin fragments between 300–400 bp. 100μl of chromatin preparation was kept as the input (total chromatin). The rest (200μl) was submitted to phenol-chloroform extraction and the aqueous phase containing the decompacted chromatin (FAIRE chromatin) was kept. Input and FAIRE DNA were purified with the Ipure kit (Diagenode) in 150μl of water and 4μl were used *per* qPCR reaction. qPCR experiments were carried out in a CFX96 system (Biorad) using SsoFast EvaGreen Supermix (Biorad). Primers used are listed in S1 Table. Data were normalized against input chromatin. Results present the mean of three independent experiments.

### Statistical analyses

To analyse the effect of temperature on A5, A6 and A7 pigmentation, we performed a one-way ANOVA (or Welch’s ANOVA when variances were heterogeneous) with temperature as factor. To analyse the effect of *t* (Fig 4) or *trx* (Fig 8D) on pigmentation plasticity, we used a two-way ANOVA with genotype and temperature as factors. The variable analysed was the first component of a Principal Component Analysis of pigmentation in A5, A6 and A7 conducted on correlations, which captures more than 95% of total variation in both cases. ANOVAs and Welch’s ANOVA were performed using the OpenStat software (W.G. Miller, http://statprogramsplus.com/OpenStatMain.htm). Normality of the residual distributions was checked with a Shapiro-Wilk test (Anastats; http://anastats.fr).

For t-tests, we checked first homogeneity of variance using a Levene Test (Anastats; http://anastats.fr) and then used the appropriate option of t-test.

## Supporting Information

S1 Fig *ebony* is epistatic over *tan*. Down-regulation or up-regulation of *tan* in the abdominal dorsal domain using the *pnr-Gal4* driver and *UAS-RNAi-t* or *UAS-t* transgene, respectively, in a wild-type (above) or *ebony* (*e*^*1*^, below) mutant background at 25°C. In a wild-type background, *tan* down-regulation strongly reduced pigmentation in the 6^th^ abdominal segment (*), whereas *tan* over-expression increased melanin production in all segments. In contrast, the modulation of *tan* expression had no effect on pigmentation in an *ebony* mutant background.
(TIF)

S2 Fig Reaction norms of *t*^*d07784*^ mutants. Reaction norms of pigmentation in A5, A6 and A7 abdominal segments of *t*^*d07784*^ or *w*^*1118*^ females (n = 10 per condition).
(TIF)

S3 Fig Expression at 18°C and 29°C of the pigmentation gene *ebony* in the abdominal epidermis of females. (A) Analysis by *in situ* hybridization of *ebony* expression pattern in the abdominal epidermis of freshly hatched *w*^*1118*^ females. Left and middle: *ebony* antisense probe at 18°C and 29°C. Right: Sense *ebony* control probe at 29°C. Note the similar expression patterns of *ebony* at 18°C and 29°C in A5, A6 and A7 segments. (B, C) Expression of *ebony* at 18°C and 29°C monitored with the *ebony-nEGFP* transgene in the abdominal epidermis of freshly hatched females. (B) nEGFP fluorescence in abdominal epidermes. At 29°C, the fluorescence on the left part of the tissue is from the pleura and the bright region in the bottom marked by an asterisk is a part of the genitalia. (C) Quantification of nEGFP fluorescence in A5, A6 and A7 hemi-tergites at 18° and 29°C (n = 10 *per* temperature). In A6 and A7, nEGFP intensity is higher at 29°C that at 18°C (t-test; **: p<0.01; ***: p<0.001).
(TIF)

S4 Fig Expression of *vestigial* (*vg*) and *CG12119* in the abdominal posterior epidermis of females grown at 18°C or 29°C. RT-qPCR experiments showing that expression of *vg* (A) and *CG12119* (B) is not significantly modulated by temperature. Note that *vg* is expressed at a very low level. In A and B, n = 3; error bars: standard deviations.
(TIF)

S5 Fig Analysis by ChIP of chromatin structure of *t* and a neighbouring gene in abdominal epidermis of females grown at 18°C or 29°C. (A) PanH3 IP signal normalized to input signal for *VG01*, *t_MSE*, *t-TSS-down*, *t-ex2*, *CG12119-TSS-down* and *NC*. (B) H3K27ac IP signal normalized to input signal for *VG01* and *t-MSE*. (C) H3K4me3 IP signal normalized to panH3 IP for *t-TSS-down*, *t-ex2*, *CG12119-TSS-down* and *NC*. In A, B, C, n = 3, error bars: standard deviations.
(TIF)

S6 Fig *trx* is involved in the regulation of several pigmentation genes in adult female abdominal epidermis. Quantification of pigmentation gene expression in posterior abdominal epidermes (segments A5, A6 and A7) from young *y-Gal4*>*UAS-RNAi*-*trx* and *y-Gal4>UAS-RNAi-GFP* females grown at 18°C (pools of 30 epidermes, n = 2, error bars: standard deviations; gene expressions in *y-Gal4*>*UAS-RNAi*-*trx* females have been normalized on gene expressions in *y-Gal4>UAS-RNAi-GFP* females). (t-test: *: p<0.05; ***: p<0.001).
(TIF)

S7 Fig Reaction norms of *trx* heterozygote mutants. Reaction norms of pigmentation in A5, A6 and A7 abdominal segments of *trx*^*j14A6*^ heterozygous and *w*^*1118*^ females (n = 30 per condition).
(TIF)

S1 Table Primers used in this study. (DOCX)

## References

[1] Pigliucci M. Phenotypic Plasticity, Beyond Nature and Nurture. Baltimore and London; 2001.

[2] Via S, Gomulkievicz R, de Jong G, Scheiner SM, Schlichting CD, van Tienderen PH. Adaptive phenotypic plasticity: consensus and controversy. TREE. 1995;10: 212–217. 21237012 10.1016/s0169-5347(00)89061-8

[3] Espinosa-Soto C, Martin OC, Wagner A. Phenotypic plasticity can facilitate adaptive evolution in gene regulatory circuits. BMC Evol Biol. 2011;11: 5 10.1186/1471-2148-11-5 21211007 PMC3024936

[4] Fierst JL. A history of phenotypic plasticity accelerates adaptation to a new environment. J Evol Biol. 2011;24: 1992–2001. 10.1111/j.1420-9101.2011.02333.x 21649767

[5] Moczek AP, Sultan S, Foster S, Ledon-Rettig C, Dworkin I, Nijhout HF, et al The role of developmental plasticity in evolutionary innovation. Proc Biol Sci. 2011; 10.1098/rspb.2011.0971 PMC3145196 21676977

[6] West-Eberhard MJ. Developmental plasticity and the origin of species differences. Proc Natl Acad Sci U A. 2005;102 Suppl 1: 6543–9. 10.1073/pnas.0501844102 PMC1131862 15851679

[7] Waddington CH. Selection of the genetic basis for an acquired character. Nature. 1952;169: 278. 10.1038/169278a0 14910749

[8] Waddington CH. Canalization of development and genetic assimilation of acquired characters. Nature. 1959;183: 1654–5. 13666847 10.1038/1831654a0

[9] Susoy V, Ragsdale EJ, Kanzaki N, Sommer RJ. Rapid diversification associated with a macroevolutionary pulse of developmental plasticity. eLife. 2015;4 10.7554/eLife.05463 PMC4357287 25650739

[10] Zhou S, Campbell TG, Stone EA, Mackay TF, Anholt RR. Phenotypic plasticity of the Drosophila transcriptome. PLoS Genet. 2012;8: e1002593 10.1371/journal.pgen.1002593 22479193 PMC3315458

[11] Kucharski R, Maleszka J, Foret S, Maleszka R. Nutritional control of reproductive status in honeybees via DNA methylation. Science. 2008;319: 1827–30. 10.1126/science.1153069 18339900

[12] Simola DF, Graham RJ, Brady CM, Enzmann BL, Desplan C, Ray A, et al Epigenetic (re)programming of caste-specific behavior in the ant Camponotus floridanus. Science. 2016;351 10.1126/science.aac6633 PMC5057185 26722000

[13] Leung A, Parks BW, Du J, Trac C, Setten R, Chen Y, et al Open chromatin profiling in mice livers reveals unique chromatin variations induced by high fat diet. J Biol Chem. 2014; 10.1074/jbc.M114.581439 PMC4156056 25006255

[14] Gibert P, Moreteau B, David JR. Developmental constraints on an adaptive plasticity: reaction norms of pigmentation in adult segments of Drosophila melanogaster. Evol Dev. 2000;2: 249–60. 11252554 10.1046/j.1525-142x.2000.00064.x

[15] Camino EM, Butts JC, Ordway A, Vellky JE, Rebeiz M, Williams TM. The evolutionary origination and diversification of a dimorphic gene regulatory network through parallel innovations in cis and trans. PLoS Genet. 2015;11: e1005136 10.1371/journal.pgen.1005136 25835988 PMC4383587

[16] Williams TM, Selegue JE, Werner T, Gompel N, Kopp A, Carroll SB. The regulation and evolution of a genetic switch controlling sexually dimorphic traits in Drosophila. Cell. 2008;134: 610–23. 10.1016/j.cell.2008.06.052 18724934 PMC2597198

[17] Jeong S, Rebeiz M, Andolfatto P, Werner T, True J, Carroll SB. The evolution of gene regulation underlies a morphological difference between two Drosophila sister species. Cell. 2008;132: 783–93. 10.1016/j.cell.2008.01.014 18329365

[18] Jeong S, Rokas A, Carroll SB. Regulation of body pigmentation by the Abdominal-B Hox protein and its gain and loss in Drosophila evolution. Cell. 2006;125: 1387–99. 16814723 10.1016/j.cell.2006.04.043

[19] Wittkopp PJ, Stewart EE, Arnold LL, Neidert AH, Haerum BK, Thompson EM, et al Intraspecific polymorphism to interspecific divergence: genetics of pigmentation in Drosophila. Science. 2009;326: 540–4. 10.1126/science.1176980 19900891

[20] Bastide H, Betancourt A, Nolte V, Tobler R, Stobe P, Futschik A, et al A Genome-Wide, Fine-Scale Map of Natural Pigmentation Variation in Drosophila melanogaster. PLoS Genet. 2013;9: e1003534 10.1371/journal.pgen.1003534 23754958 PMC3674992

[21] Dembeck LM, Huang W, Magwire MM, Lawrence F, Lyman RF, Mackay TFC. Genetic Architecture of Abdominal Pigmentation in Drosophila melanogaster. PLoS Genet. 2015;11: e1005163 10.1371/journal.pgen.1005163 25933381 PMC4416719

[22] Rogers WA, Salomone JR, Tacy DJ, Camino EM, Davis KA, Rebeiz M, et al Recurrent modification of a conserved cis-regulatory element underlies fruit fly pigmentation diversity. PLoS Genet. 2013;9: e1003740 10.1371/journal.pgen.1003740 24009528 PMC3757066

[23] Ordway AJ, Hancuch KN, Johnson W, Wiliams TM, Rebeiz M. The expansion of body coloration involves coordinated evolution in cis and trans within the pigmentation regulatory network of Drosophila prostipennis. Dev Biol. 2014;392: 431–40. 10.1016/j.ydbio.2014.05.023 24907418

[24] Hoffmann AA. Physiological climatic limits in Drosophila: patterns and implications. J Exp Biol. 2010;213: 870–80. 10.1242/jeb.037630 20190112

[25] Gibert JM, Peronnet F, Schlotterer C. Phenotypic Plasticity in Drosophila Pigmentation Caused by Temperature Sensitivity of a Chromatin Regulator Network. PLoS Genet. 2007;3: e30 17305433 10.1371/journal.pgen.0030030 PMC1797818

[26] Cooley AM, Shefner L, McLaughlin WN, Stewart EE, Wittkopp PJ. The ontogeny of color: developmental origins of divergent pigmentation in Drosophila americana and D. novamexicana. Evol Dev. 2012;14: 317–25. 10.1111/j.1525-142X.2012.00550.x 22765203 PMC3402224

[27] Martin A, Orgogozo V. The Loci of repeated evolution: a catalog of genetic hotspots of phenotypic variation. Evolution. 2013;67: 1235–50. 10.1111/evo.12081 23617905

[28] Riedel F, Vorkel D, Eaton S. Megalin-dependent yellow endocytosis restricts melanization in the Drosophila cuticle. Development. 2011;138: 149–58. 10.1242/dev.056309 21138977

[29] Wittkopp PJ, Carroll SB, Kopp A. Evolution in black and white: genetic control of pigment patterns in Drosophila. Trends Genet. 2003;19: 495–504. 12957543 10.1016/S0168-9525(03)00194-X

[30] Rebeiz M, Ramos-Womack M, Jeong S, Andolfatto P, Werner T, True J, et al Evolution of the tan locus contributed to pigment loss in Drosophila santomea: a response to Matute et al. Cell. 2009;139: 1189–1196. 10.1016/j.cell.2009.11.004 20005811

[31] True JR, Yeh SD, Hovemann BT, Kemme T, Meinertzhagen IA, Edwards TN, et al Drosophila tan Encodes a Novel Hydrolase Required in Pigmentation and Vision. PLoS Genet. 2005;1: e63 16299587 10.1371/journal.pgen.0010063 PMC1285064

[32] Calleja M, Herranz H, Estella C, Casal J, Lawrence P, Simpson P, et al Generation of medial and lateral dorsal body domains by the pannier gene of Drosophila. Development. 2000;127: 3971–80. 10952895 10.1242/dev.127.18.3971

[33] Dietzl G, Chen D, Schnorrer F, Su KC, Barinova Y, Fellner M, et al A genome-wide transgenic RNAi library for conditional gene inactivation in Drosophila. Nature. 2007;448: 151–6. 10.1038/nature05954 17625558

[34] Rebeiz M, Pool JE, Kassner VA, Aquadro CF, Carroll SB. Stepwise modification of a modular enhancer underlies adaptation in a Drosophila population. Science. 2009;326: 1663–7. 10.1126/science.1178357 20019281 PMC3363996

[35] Song L, Zhang Z, Grasfeder LL, Boyle AP, Giresi PG, Lee BK, et al Open chromatin defined by DNaseI and FAIRE identifies regulatory elements that shape cell-type identity. Genome Res. 2011;21: 1757–67. 10.1101/gr.121541.111 21750106 PMC3202292

[36] Thomas S, Li X-Y, Sabo PJ, Sandstrom R, Thurman RE, Canfield TK, et al Dynamic reprogramming of chromatin accessibility during Drosophila embryo development. Genome Biol. 2011;12: R43 10.1186/gb-2011-12-5-r43 21569360 PMC3219966

[37] Giresi PG, Kim J, McDaniell RM, Iyer VR, Lieb JD. FAIRE (Formaldehyde-Assisted Isolation of Regulatory Elements) isolates active regulatory elements from human chromatin. Genome Res. 2007;17: 877–85. 10.1101/gr.5533506 17179217 PMC1891346

[38] McKay DJ, Lieb JD. A common set of DNA regulatory elements shapes Drosophila appendages. Dev Cell. 2013;27: 306–318. 10.1016/j.devcel.2013.10.009 24229644 PMC3866527

[39] Bonn S, Zinzen RP, Girardot C, Gustafson EH, Perez-Gonzalez A, Delhomme N, et al Tissue-specific analysis of chromatin state identifies temporal signatures of enhancer activity during embryonic development. Nat Genet. 2012;44: 148–156. 10.1038/ng.1064 22231485

[40] Mito Y, Henikoff JG, Henikoff S. Genome-scale profiling of histone H3.3 replacement patterns. Nat Genet. 2005;37: 1090–1097. 10.1038/ng1637 16155569

[41] Yin H, Sweeney S, Raha D, Snyder M, Lin H. A high-resolution whole-genome map of key chromatin modifications in the adult Drosophila melanogaster. PLoS Genet. 2011;7: e1002380 10.1371/journal.pgen.1002380 22194694 PMC3240582

[42] Mohan M, Herz H-M, Smith ER, Zhang Y, Jackson J, Washburn MP, et al The COMPASS family of H3K4 methylases in Drosophila. Mol Cell Biol. 2011;31: 4310–4318. 10.1128/MCB.06092-11 21875999 PMC3209330

[43] Petruk S, Sedkov Y, Smith S, Tillib S, Kraevski V, Nakamura T, et al Trithorax and dCBP acting in a complex to maintain expression of a homeotic gene. Science. 2001;294: 1331–4. 11701926 10.1126/science.1065683

[44] Herz HM, Mohan M, Garruss AS, Liang K, Takahashi YH, Mickey K, et al Enhancer-associated H3K4 monomethylation by Trithorax-related, the Drosophila homolog of mammalian Mll3/Mll4. Genes Dev. 2012;26: 2604–20. 10.1101/gad.201327.112 23166019 PMC3521626

[45] Hallson G, Hollebakken RE, Li T, Syrzycka M, Kim I, Cotsworth S, et al dSet1 is the main H3K4 di- and tri-methyltransferase throughout Drosophila development. Genetics. 2012;190: 91–100. 10.1534/genetics.111.135863 22048023 PMC3249358

[46] Tie F, Banerjee R, Saiakhova AR, Howard B, Monteith KE, Scacheri PC, et al Trithorax monomethylates histone H3K4 and interacts directly with CBP to promote H3K27 acetylation and antagonize Polycomb silencing. Dev Camb Engl. 2014;141: 1129–1139. 10.1242/dev.102392 PMC3929413 24550119

[47] Smith ST, Petruk S, Sedkov Y, Cho E, Tillib S, Canaani E, et al Modulation of heat shock gene expression by the TAC1 chromatin-modifying complex. Nat Cell Biol. 2004;6: 162–167. 10.1038/ncb1088 14730313

[48] Chen J, Nolte V, Schlötterer C. Temperature-Related Reaction Norms of Gene Expression: Regulatory Architecture and Functional Implications. Mol Biol Evol. 2015;32: 2393–2402. 10.1093/molbev/msv120 25976350 PMC4540970

[49] Crocker J, Abe N, Rinaldi L, McGregor AP, Frankel N, Wang S, et al Low affinity binding site clusters confer hox specificity and regulatory robustness. Cell. 2015;160: 191–203. 10.1016/j.cell.2014.11.041 25557079 PMC4449256

[50] Kharchenko PV, Alekseyenko AA, Schwartz YB, Minoda A, Riddle NC, Ernst J, et al Comprehensive analysis of the chromatin landscape in Drosophila melanogaster. Nature. 2011;471: 480–5. 10.1038/nature09725 21179089 PMC3109908

[51] Börsch-Haubold AG, Montero I, Konrad K, Haubold B. Genome-wide quantitative analysis of histone H3 lysine 4 trimethylation in wild house mouse liver: environmental change causes epigenetic plasticity. PloS One. 2014;9: e97568 10.1371/journal.pone.0097568 24849289 PMC4029994

[52] Zong W, Zhong X, You J, Xiong L. Genome-wide profiling of histone H3K4-tri-methylation and gene expression in rice under drought stress. Plant Mol Biol. 2013;81: 175–188. 10.1007/s11103-012-9990-2 23192746

[53] van Dijk K, Ding Y, Malkaram S, Riethoven J-JM, Liu R, Yang J, et al Dynamic changes in genome-wide histone H3 lysine 4 methylation patterns in response to dehydration stress in Arabidopsis thaliana. BMC Plant Biol. 2010;10: 238 10.1186/1471-2229-10-238 21050490 PMC3095321

[54] Weiner A, Chen HV, Liu CL, Rahat A, Klien A, Soares L, et al Systematic dissection of roles for chromatin regulators in a yeast stress response. PLoS Biol. 2012;10: e1001369 10.1371/journal.pbio.1001369 22912562 PMC3416867

[55] Fedorka KM, Copeland EK, Winterhalter WE. Seasonality influences cuticle melanization and immune defense in a cricket: support for a temperature-dependent immune investment hypothesis in insects. J Exp Biol. 2013;216: 4005–10. 10.1242/jeb.091538 23868839

[56] Michie LJ, Mallard F, Majerus ME, Jiggins FM. Melanic through nature or nurture: genetic polymorphism and phenotypic plasticity in Harmonia axyridis. J Evol Biol. 2010;23: 1699–707. 10.1111/j.1420-9101.2010.02043.x 20626543

[57] Daniels EV, Murad R, Mortazavi A, Reed RD. Extensive transcriptional response associated with seasonal plasticity of butterfly wing patterns. Mol Ecol. 2014; 10.1111/mec.12988 PMC4545284 25369871

[58] Yassin A, Bastide H, Chung H, Veuille M, David JR, Pool JE. Ancient balancing selection at tan underlies female colour dimorphism in Drosophila erecta. Nat Commun. 2016;7: 10400 10.1038/ncomms10400 26778363 PMC4735637

[59] Sicard A, Thamm A, Marona C, Lee YW, Wahl V, Stinchcombe JR, et al Repeated evolutionary changes of leaf morphology caused by mutations to a homeobox gene. Curr Biol. 2014;24: 1880–6. 10.1016/j.cub.2014.06.061 25127212

[60] Gindhart JG, Kaufman TC. Identification of Polycomb and trithorax group responsive elements in the regulatory region of the Drosophila homeotic gene Sex combs reduced. Genetics. 1995;139: 797–814. 7713433 10.1093/genetics/139.2.797 PMC1206382

[61] Bainbridge SP, Bownes M. Staging the metamorphosis of Drosophila melanogaster. J Embryol Exp Morphol. 1981;66: 57–80. 6802923

[62] Pfaffl MW. A new mathematical model for relative quantification in real-time RT-PCR. Nucleic Acids Res. 2001;29: e45 11328886 10.1093/nar/29.9.e45 PMC55695

[63] Vandesompele J, De Preter K, Pattyn F, Poppe B, Van Roy N, De Paepe A, et al Accurate normalization of real-time quantitative RT-PCR data by geometric averaging of multiple internal control genes. Genome Biol. 2002;3: RESEARCH0034 12184808 10.1186/gb-2002-3-7-research0034 PMC126239

[64] Coléno-Costes A, Jang SM, de Vanssay A, Rougeot J, Bouceba T, Randsholt NB, et al New partners in regulation of gene expression: the enhancer of Trithorax and Polycomb Corto interacts with methylated ribosomal protein l12 via its chromodomain. PLoS Genet. 2012;8: e1003006 10.1371/journal.pgen.1003006 23071455 PMC3469418

