# Supplementary figures and images for: Phenotypic Plasticity through Transcriptional Regulation of the Evolutionary Hotspot Gene tan in Drosophila melanogaster
Source: PLoS Genet. 2016 Aug 10;12(8):e1006218. doi: 10.1371/journal.pgen.1006218 (PMC4980059; doi:10.1371/journal.pgen.1006218)

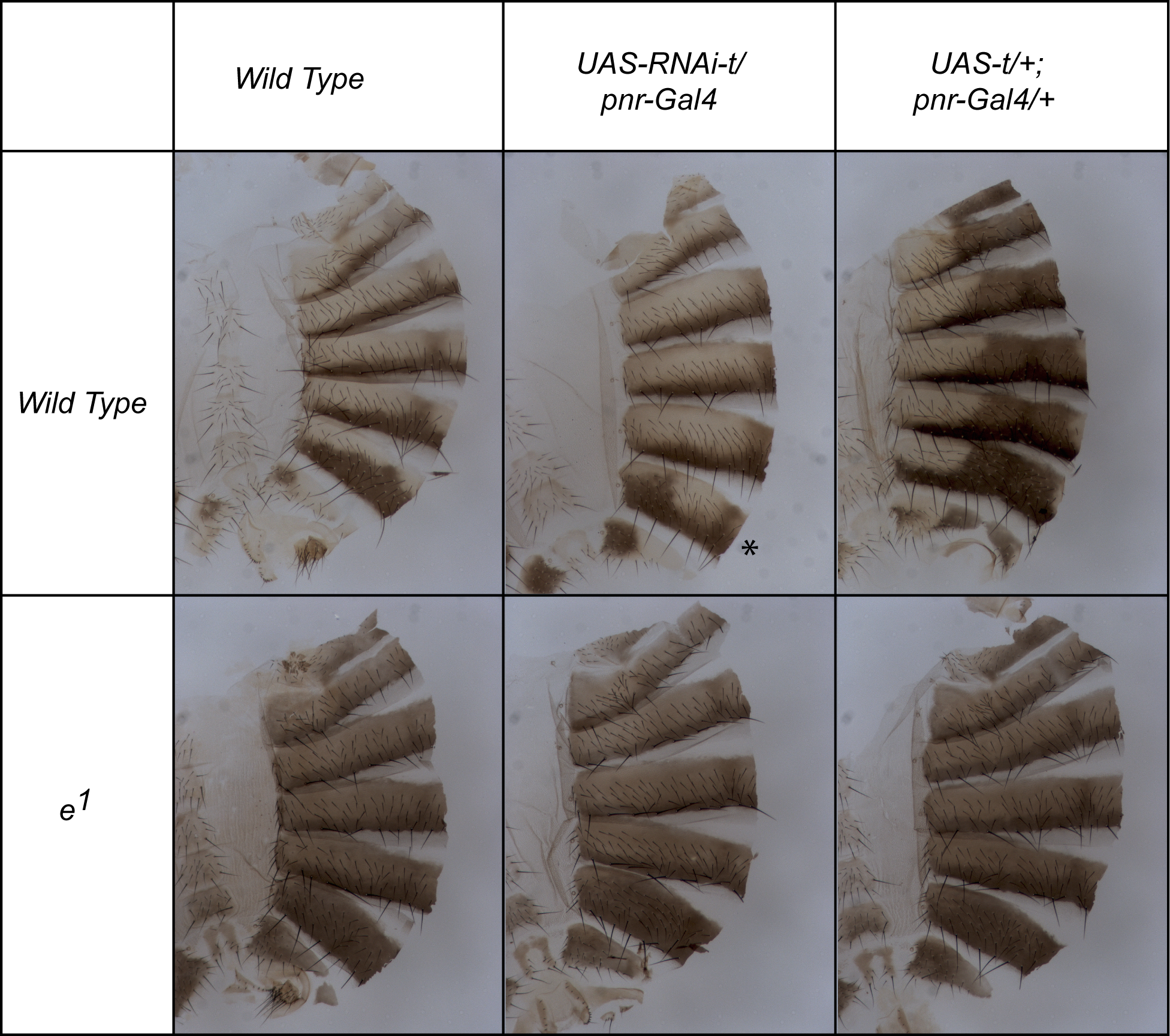

Supplement: S1 Fig — Down-regulation or up-regulation of tan in the abdominal dorsal domain using the pnr-Gal4 driver and UAS-RNAi-t or UAS-t transgene, respectively, in a wild-type (above) or ebony (e1, below) mutant background at 25°C. In a wild-type background, tan down-regulation strongly reduced pigmentation in the 6th abdominal segment (*), whereas tan over-expression increased melanin production in all segments. In contrast, the modulation of tan expression had no effect on pigmentation in an ebony mutant background. (TIF) [file pgen.1006218.s001.tif]

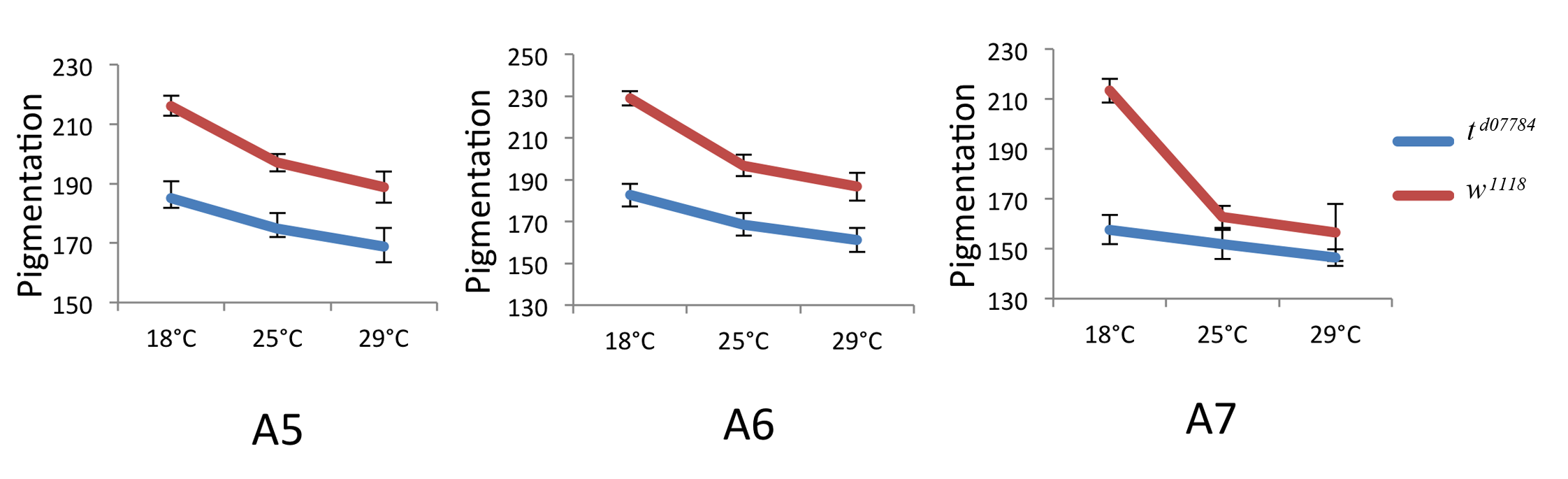

Supplement: S2 Fig — Reaction norms of pigmentation in A5, A6 and A7 abdominal segments of td07784 or w1118 females (n = 10 per condition). (TIF) [file pgen.1006218.s002.tif]

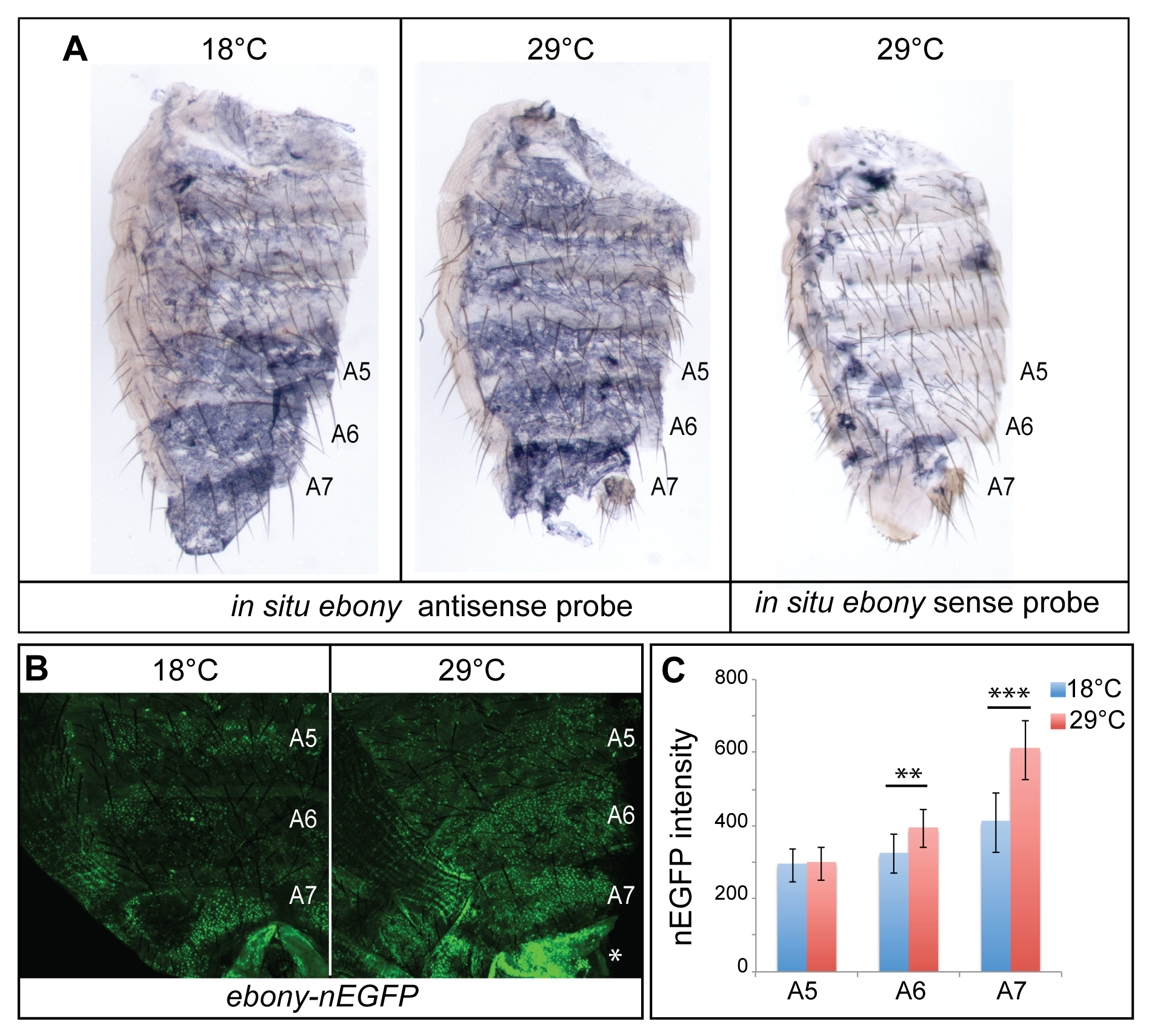

Supplement: S3 Fig — (A) Analysis by in situ hybridization of ebony expression pattern in the abdominal epidermis of freshly hatched w1118 females. Left and middle: ebony antisense probe at 18°C and 29°C. Right: Sense ebony control probe at 29°C. Note the similar expression patterns of ebony at 18°C and 29°C in A5, A6 and A7 segments. (B, C) Expression of ebony at 18°C and 29°C monitored with the ebony-nEGFP transgene in the abdominal epidermis of freshly hatched females. (B) nEGFP fluorescence in abdominal epidermes. At 29°C, the fluorescence on the left part of the tissue is from the pleura and the bright region in the bottom marked by an asterisk is a part of the genitalia. (C) Quantification of nEGFP fluorescence in A5, A6 and A7 hemi-tergites at 18° and 29°C (n = 10 per temperature). In A6 and A7, nEGFP intensity is higher at 29°C that at 18°C (t-test; **: p<0.01; ***: p<0.001). (TIF) [file pgen.1006218.s003.tif]

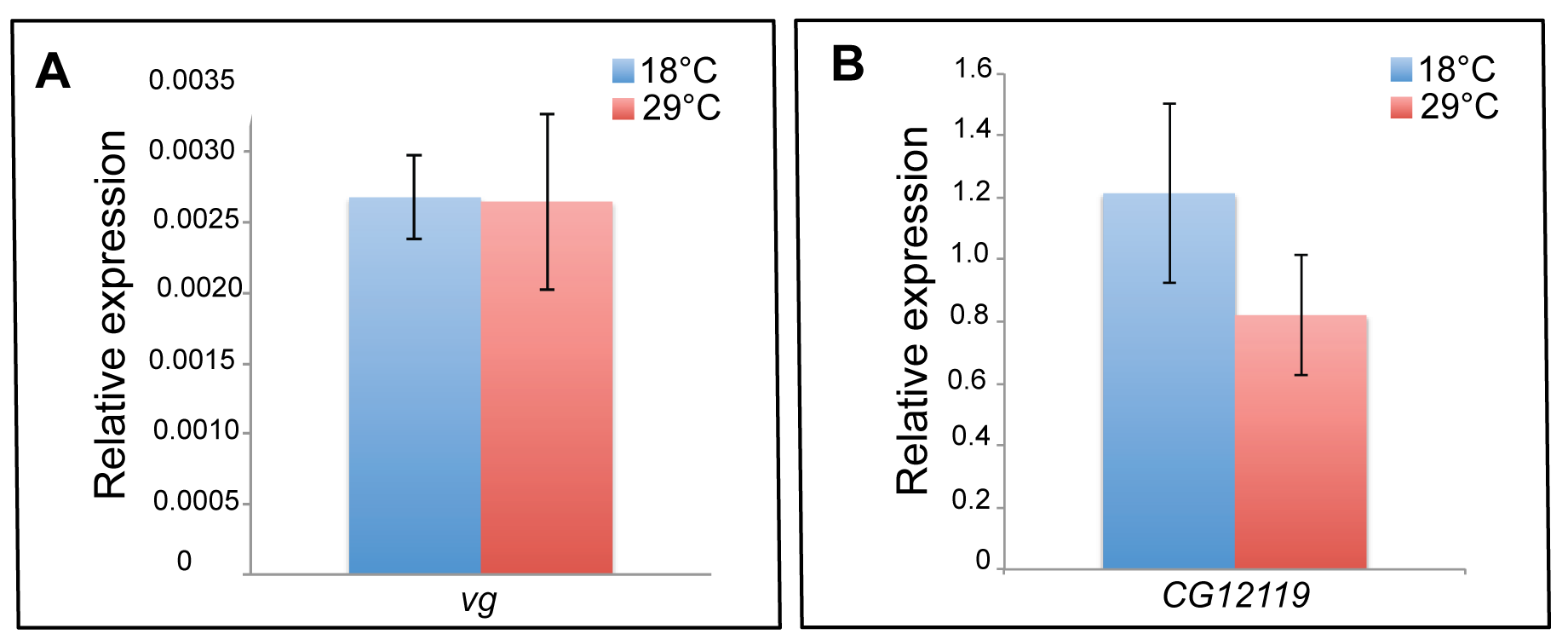

Supplement: S4 Fig — RT-qPCR experiments showing that expression of vg (A) and CG12119 (B) is not significantly modulated by temperature. Note that vg is expressed at a very low level. In A and B, n = 3; error bars: standard deviations. (TIF) [file pgen.1006218.s004.tif]

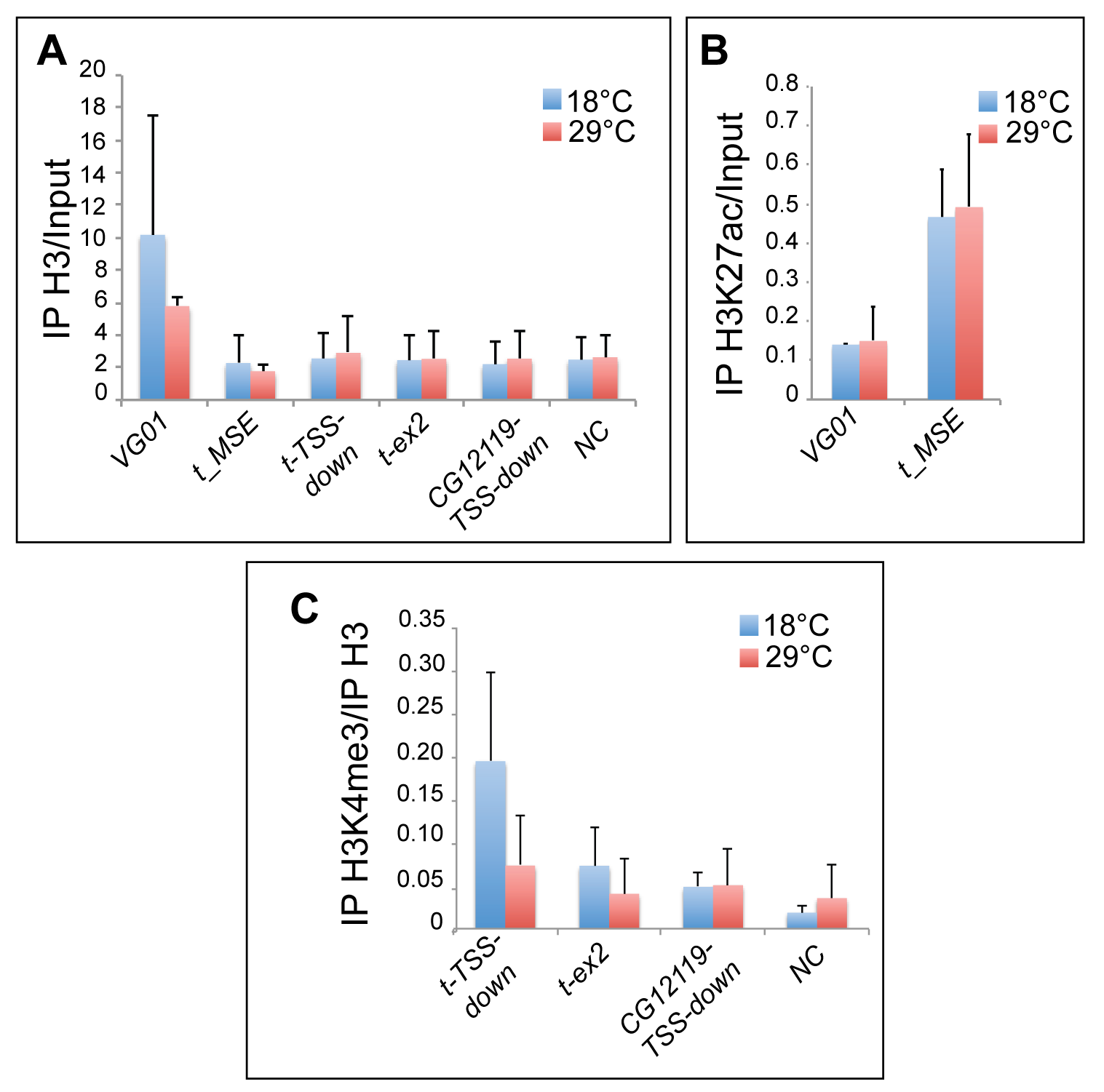

Supplement: S5 Fig — (A) PanH3 IP signal normalized to input signal for VG01, t_MSE, t-TSS-down, t-ex2, CG12119-TSS-down and NC. (B) H3K27ac IP signal normalized to input signal for VG01 and t-MSE. (C) H3K4me3 IP signal normalized to panH3 IP for t-TSS-down, t-ex2, CG12119-TSS-down and NC. In A, B, C, n = 3, error bars: standard deviations. (TIF) [file pgen.1006218.s005.tif]

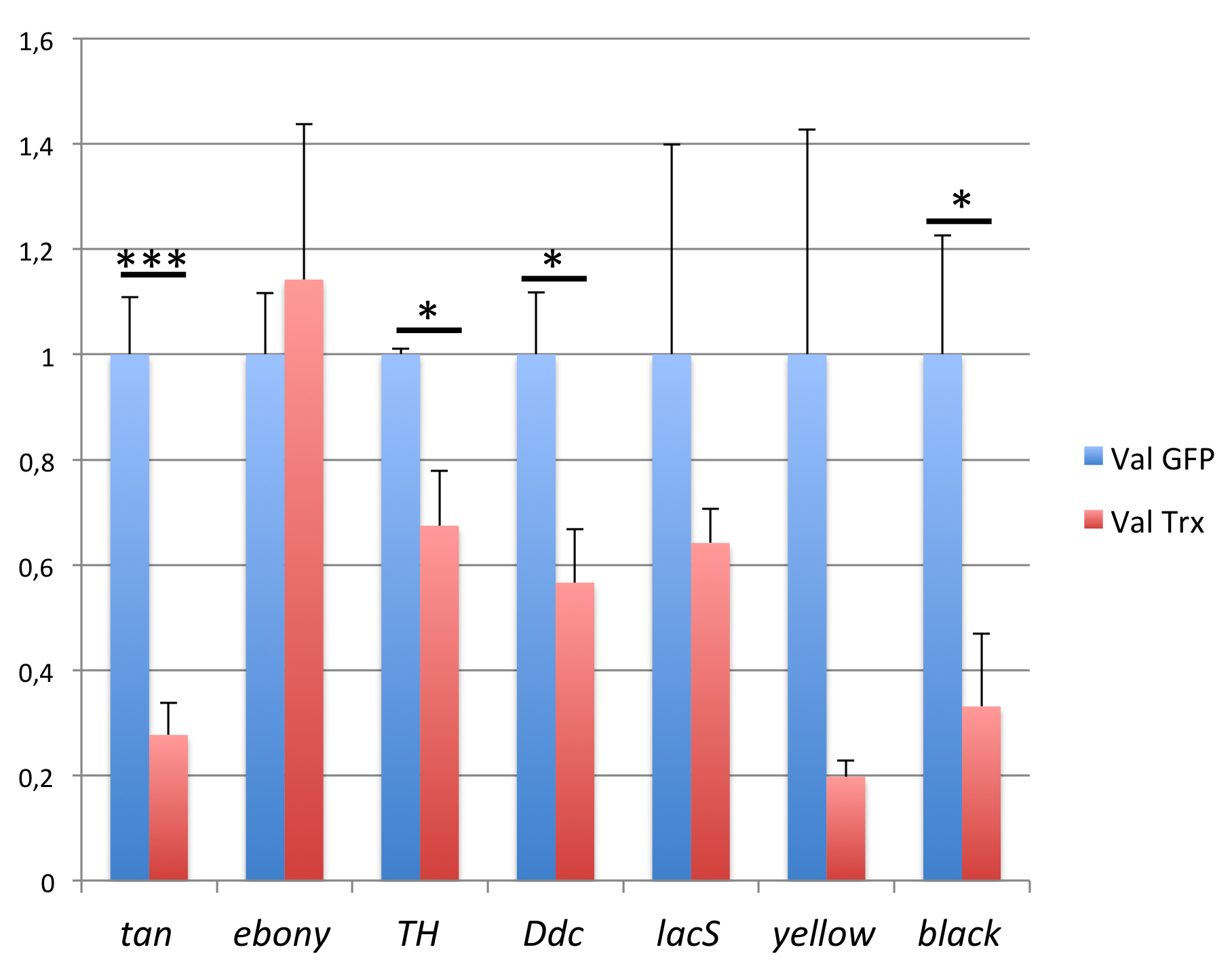

Supplement: S6 Fig — Quantification of pigmentation gene expression in posterior abdominal epidermes (segments A5, A6 and A7) from young y-Gal4>UAS-RNAi-trx and y-Gal4>UAS-RNAi-GFP females grown at 18°C (pools of 30 epidermes, n = 2, error bars: standard deviations; gene expressions in y-Gal4>UAS-RNAi-trx females have been normalized on gene expressions in y-Gal4>UAS-RNAi-GFP females). (t-test: *: p<0.05; ***: p<0.001). (TIF) [file pgen.1006218.s006.tif]

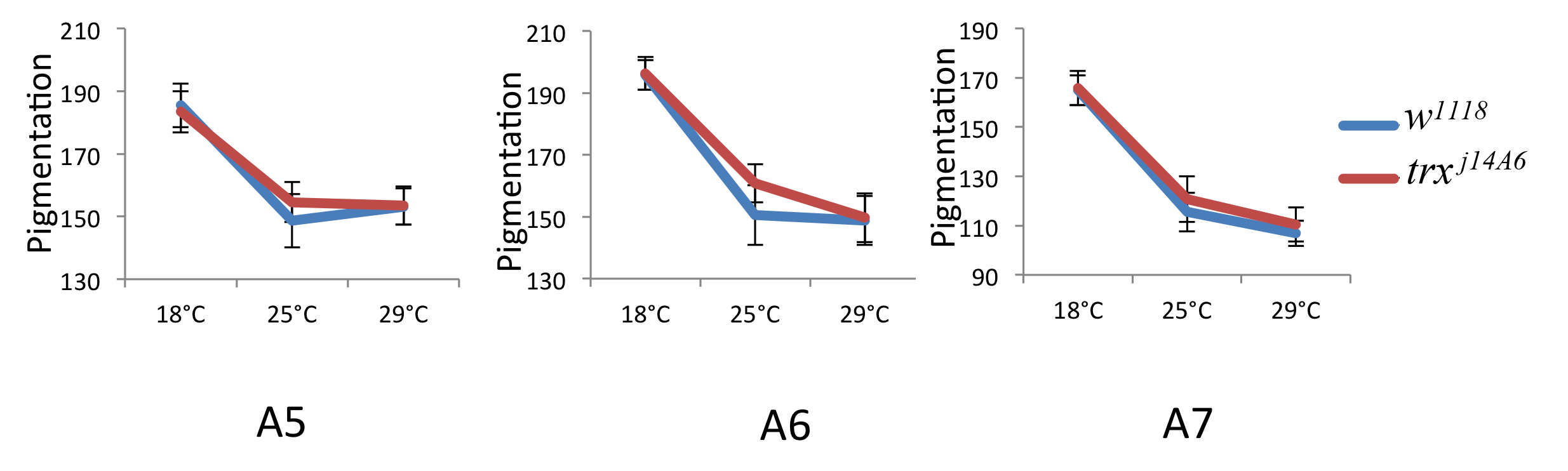

Supplement: S7 Fig — Reaction norms of pigmentation in A5, A6 and A7 abdominal segments of trxj14A6 heterozygous and w1118 females (n = 30 per condition). (TIF) [file pgen.1006218.s007.tif]
