# Supplementary material for: Phenotypic Plasticity through Transcriptional Regulation of the Evolutionary Hotspot Gene tan in Drosophila melanogaster
Source: PLoS Genet. 2016 Aug 10;12(8):e1006218. doi: 10.1371/journal.pgen.1006218 (PMC4980059; doi:10.1371/journal.pgen.1006218)
Supplement: S1 Table — (DOCX) [file pgen.1006218.s008.docx]

|  |  |  |  |
| --- | --- | --- | --- |
| Gene | Primer | Sequence (5'->3') | Experiment |
| *tan* | TS-F | GACCTACTTGCCGCTCTACC |  |
|  | TS-R | GATCCGCCAGGAAGGTGAAA | *in situ* |
| *ebony* | ES-F | GATCAGGCGTCTCGTACTGG | hybridizations |
|  | ES-R | TGACCAGCATGGTAGCACAG |  |
| *tan* | T-F | ACAGGGAAATGGTCCTGGCACTGA |  |
|  | T-R | TTTACTGCGTCTGGCGGGAGAA |  |
| *ebony* | E-F | CTGGAATGTGCTGGTGGAG |  |
|  | E-R | AGACCCTTGGGCAGGTAGTT |  |
| *TH* | TH-F | TGTGTATGTTTGGGTGGTGGGGGT |  |
|  | TH-R | AGTTGCGACGCCCGTTCTAAGT |  |
| *Ddc* | D-F | AGCATCTTGCCCAGCCAATCCA |  |
|  | D-R | TGAGTGGAGCGATTGCCTGCAT |  |
| *yellow* | Y-F | AGTGTGGTCGGCTGTGGGTTTT |  |
|  | Y-R | TGGATTTGTGTCCACGCCAGGT |  |
| *laccase2* | Lacs-F | CAATAGCCTTGGGTCTGGA |  |
|  | Lacs-R | TGGATAACGTGCAGAGGTCG | RT-qPCR |
| *black* | Black-F | TGCCAAGCCGCTGATTATCT |  |
|  | Black-R | TGTTTCTCCAGGTCGCTCAG |  |
| *vestigial* | Vg-F | CACCAGGTTAGCGACTTGT |  |
|  | Vg-R | GCGTGGGCATAGGAACCATA |  |
| *CG12119* | 12119-F | CACAGATCGGACGAACGACA |  |
|  | 12119-R | CGTGATTGACGACGCCAAAG |  |
| *rp49* | RP49-F | CCGCTTCAAGGGACAGTATC |  |
|  | RP49-R | GACAATCTCCTTGCGCTTCT |  |
| *Act5C* | Act5C-F  Act5C-R | AGCGCGGTTACTCTTTCACCAC  GTGGCCATCTCCTGCTCAAAGT |  |
| *eIF2* | eIF2-F | TCGCATCAACCTGATAGCAC |  |
|  | eIF2-R | ATCGTACTCGCTGGTCTTGG |  |
| *vestigial* | VG01-F | CGACGGAAGAGACAAACCCA |  |
|  | VG01-R | CGTCTCCATCTTCGCTCCAT |  |
| *tan* | t_MSE-F | TTTGTTTCAACTCAATCCTAGCAG |  |
|  | t_MSE-R | TTTCAAGTGGTCTTGGTGCT |  |
| *tan* | t-TSS-up-F | CACGCTGTGTAGTTTGGGTT |  |
|  | t-TSS-up-R | AGCTGATCTCTGGACTTGCT |  |
| *tan* | t-TSS-down-F | CCTGGTGTAGAGCACTGGC |  |
|  | t-TSS-down-R | CCGAACCCCGAACCCATTTT |  |
| *tan* | t-ex2-F | GGTAGAGCGGCAAGTAGGTC | FAIRE/ChIP |
|  | t-ex2-R | TCGCACATTTGGGTCCATGA |  |
| *CG12119* | CG12119-TSS-up-F | AGTGCCAGTTGAATGTACGAA |  |
|  | CG12119-TSS-up-R | ATATCTGTACATCCGCCGCC |  |
| *CG12119* | CG12119-TSS-down-F | GCCAGAAACGAACGCCATAC |  |
|  | CG12119-TSS-down-R | CATCGTGACCACCACCGATT |  |
| Negative control | NC-F | CGGTTGACTTTTCGCACCTG |  |
|  | NC-R | CTGGCAACTTGCATCGCATT |  |

S1 Table: Sequences of primers used.
